# Multifaceted insights into the environmental adaptability of *Arnebia guttata* under drought stress

**DOI:** 10.3389/fpls.2024.1395046

**Published:** 2024-06-13

**Authors:** Qian Liu, Haolin Liu, Min Zhang, Guoshuai Lv, Zeyuan Zhao, Xingyu Chen, Xinxin Wei, Chunhong Zhang, Minhui Li

**Affiliations:** ^1^ Central Laboratory, Inner Mongolia Autonomous Region Hospital of Traditional Chinese Medicine, Hohhot, China; ^2^ Inner Mongolia Key Laboratory of Characteristic Geoherbs Resources Protection and Utilization, Baotou Medical College, Baotou, China; ^3^ College of Pharmacy, Inner Mongolia Medical University, Hohhot, China

**Keywords:** *Arnebia guttata*, drought stress, shikonin, environmental adaptability, multiple producing areas, metabolomic profiling, transcriptomic profiling

## Abstract

**Introduction:**

Global warming has led to increased environmental stresses on plants, notably drought. This affects plant distribution and species adaptability, with some medicinal plants showing enhanced drought tolerance and increased medicinal components. In this pioneering study, we delve into the intricate tapestry of *Arnebia guttata*, a medicinal plant renowned for its resilience in arid environments. By fusing a rich historical narrative with cutting-edge analytical methodologies, this research endeavors to demystify the plant’s intricate response to drought stress, illuminating its profound implications for medicinal valorization.

**Methods:**

The methodology includes a comprehensive textual research and resource investigation of *A. guttata*, regionalization studies, field sample distribution analysis, transcriptome and metabolome profiling, rhizosphere soil microbiome analysis, and drought stress experiments. Advanced computational tools like ArcGIS, MaxEnt, and various bioinformatics software were utilized for data analysis and modeling.

**Results:**

The study identified significant genetic variations among *A. guttata* samples from different regions, correlating with environmental factors, particularly precipitation during the warmest quarter (BIO18). Metabolomic analysis revealed marked differences in metabolite profiles, including shikonin content, which is crucial for the plant’s medicinal properties. Soil microbial community analysis showed variations that could impact plant metabolism and stress response. Drought stress experiments demonstrated *A. guttata*’s resilience and its ability to modulate metabolic pathways to enhance drought tolerance.

**Discussion:**

The findings underscore the complex interplay between genetic makeup, environmental factors, and microbial communities in shaping *A. guttata*’s adaptability and medicinal value. The study provides insights into how drought stress influences the synthesis of active compounds and suggests that moderate stress could enhance the plant’s medicinal properties. Predictive modeling indicates future suitable growth areas for *A. guttata*, aiding in resource management and conservation efforts. The research contributes to the sustainable development of medicinal resources and offers strategies for improving the cultivation of *A. guttata*.

## 1 Introduction

Owing to global warming, plants experience increasing environmental stresses, such as drought. Frequent drought events lead to significant changes in the distribution of plant resources, thereby altering the adaptability and tolerance of species. Species have evolved various survival strategies in response to ecological differences. For example, wild grain adopts different methods of escaping or tolerating drought to cope with drought stress. These strategies are based on complex physiological and biochemical regulation and are significantly affected by the environment (Wang et al., 2023). However, appropriate water scarcity in the environment can be advantageous for plants, particularly certain medicinal ones. According to Liang et al. (2023), the medicinal plant *Sophora tonkinensis*, indigenous to karst areas, exhibits enhanced drought tolerance, resulting in an augmentation of medicinal components such as matrine and oxymatrine under varying soil water conditions. Furthermore, following exposure to drought conditions, the antioxidant capacity of *Melia azedarach* experienced augmentation, coupled with a notable increase in the accumulation of lipid metabolites such as fatty acids and oleamide. This implies that drought stress has the potential to enhance the nutritional value of *M. azedarach* (Dias et al., 2022). Therefore, while safeguarding the survival environment of species, exploring and judiciously applying environmental influences on medicinal plants represent a contemporary and crucial endeavour in seeking a new balance between the scarcity of wild herbal resources and the advancement of human health (Montaño López and Avalos, 2020).

*Arnebia guttata* is a perennial herb belonging to the genus *Arnebia* of the family Boraginaceae. In the Chinese Pharmacopoeia, it serves as the basic source of medicinal Arnebiae Radix. The major medicinal components of *A. guttata* include shikonin and its derivatives, which have antibacterial, anti-inflammatory, antiviral, antipyretic, liver protective, antioxidant, anti-tumour, immune regulatory, and other pharmacological effects (Guo et al., 2019). Its mechanism of action is very complex, involving cell proliferation, apoptosis, and signal transduction, making it a novel anticancer agent worth exploring, and in the normal dose range, *A. guttata* has almost no toxic side effects (Ma et al., 2020). Currently, it is the main base plant of the Chinese herbal medicine Arnebiae Radix, and the primary areas of *A. guttata* production are Inner Mongolia, Xinjiang, Ningxia, and Gansu. The high requirements for growth, large-scale artificial planting of *A. guttata* has not been performed. It is important to study the development, utilisation, and cultivation of *A. guttata* resources.

*A. guttata* grows in sandy soil and prefers sunshine. The northwest part of China, where most of its resources are distributed, is located in the interior of the continent, far from the sea. The south is blocked by plateaus and mountains, where water vapour is difficult to reach and sunlight lasts long, resulting in a dry climate (Cao et al., 2022). Previous studies on medicinal plants have indicated that drought significantly impacts the growth, development, and accumulation of bioactive compounds in these plants. This includes the synthesis and accumulation of proteins related to energy metabolism, carbohydrate metabolism, photosynthesis, stress response, and defence mechanisms (Omidi et al., 2018; Xu et al., 2021). In summary, when drought affects the development of medicinal plants, the plants themselves develop various regulatory mechanisms to adapt to the environment for coexistence and balance, which reflects the special relationship network between cell molecules and metabolites. Drought is closely related to the quality of medicinal plants. During the development and application of active components of Arnebiae Radix, the *A. guttata* will gradually become the mainstream variety. However, the correlation between the formation of medicinal components and climate remains unclear.

Field samples of *A. guttata* from Inner Mongolia, Xinjiang, and Ningxia showed that distribution is the lowest in Xinjiang, with the densest area carrying approximately 200 plants/km^2^. *A. guttata* is most densely distributed in Inner Mongolia, with approximately 1000 plants/km^2^, indicating that *A. guttata* resources in Inner Mongolia is sufficient to be used for medicinal purposes. According to a regionalisation study conducted simultaneously, the precipitation of the warmest quarter (BIO18) (May–September) was the most important climatic factor affecting the distribution of *A. guttata* (Tian et al., 2014). Significant differences in the precipitation at each sample point were noted. There is a predominance of studies on the chemical composition and pharmacological effects of *A. guttata*; however, in-depth research on its resources and components are lacking. In the present study, we utilised ArcGIS computing to investigate the resource distribution and a correlation of climatic factors for *A. guttata* under drought stress. These included the exploration of its historical use in ancient books, the distribution of national resource regionalisation, the collection and analysis of field samples from different cultivation regions, and the correlation of climatic factors with the quality of *A. guttata*. We applied metabolomic and transcriptomic analyses to uncover the potential synthesis and metabolism of active components, as well as the metabolic mechanisms under drought stress. Further validation was conducted through drought stress experiments on cultivated varieties. Additionally, we examined and analysed the soil microbiome of field samples and predicted suitable growth regions for the next 40 years. The objective of this study was to comprehensively study the distribution of *A. guttata*, explore intrinsic differences among field samples, reveal the correlation between climatic factors and the quality of *A. guttata*, elucidate the biosynthetic characteristics of active substances, and clarify the molecular response mechanisms of stress resistance in *A. guttata* under drought stress. This information provides valuable insights for enhancing its medicinal value and ensuring the sustainable development of future resources.

## 2 Materials and methods

### 2.1 Textual research and resource investigation of *A. guttata*

The historical books, literature, and website resources of Materia Medica were consulted, and relevant information on *A. guttata* was identified. The materials included, “Shen Nong’s Herbal Classic”, “The Newly Revised Materia Medica”, “Classified Materia Medica”, “Compendium of Materia Medica”, “Natural History”, “New Chinese Medicine Records”, “An Illustrated Book of Plants”, “Discussion on Varieties of Traditional Chinese Medicine”, “Sorting and Quality Study of Commonly Used Traditional Chinese Medicine Varieties”, “Chinese Materia Medica”, “Modern Traditional Chinese Medicine Commodity Review”, “Chinese Medicine Sea”, “Inner Mongolia Chinese herbal medicine”, “Error-free Mongolian Medicine Guide”, “Medicine Recognition White Crystal Mirror”; additionally, websites such as Chinese Flora were referenced.

According to the database, we visited the primary production areas, investigated the wild resources of *A. guttata*, and collected samples.

### 2.2 Study on regionalisation and analysis of field sample distribution

To predict the distribution of *A. guttata* in China, the species distribution points and census data points of the China Digital Plant Museum (CVH) (https://www.cvh.ac.cn/) were accurately collected; suspicious and repetitive sample collection points were excluded, and 49 sample collection points were determined. Twenty-eight variables, comprising 19 climatic variables, 1 topographic variable, and 8 soil variables, were selected for modelling. The spatial resolution of the data was 30 arcsec. To reduce the influence of the correlation of environmental factors on the model, the Pearson correlation coefficient (Pearson) was used to test the multicollinearity among environmental factors (Bi et al., 2022). Environmental factors with correlation coefficients greater than 0.8 were screened combined with the importance of environmental factors, and factors with a high contribution rate in the operation of the model were retained for modelling. The MaxEnt settings were as follows: the MaxEnt output mode was “Logisitc”, the maximum number of iterations of the model was 105, the convergence threshold was 0.0005, and other parameters defaulted to 10 times cross-validation. To ensure the accuracy of the model prediction, the area under the curve (AUC) was used to evaluate the model’s prediction results. Generally, an AUC value > 0.75 indicates that the prediction result is relatively good.

Based on the national census data of traditional Chinese medicine resources and our literature review of *A. guttata*, samples were collected from Inner Mongolia, Xinjiang, Ningxia, Gansu, and other provinces and regions. During collection, we maintained the integrity of the whole plant, especially the root, and dug deeply to avoid severing the roots. Immediately after uprooting, any soil that adhered to the plants was washed off with clean water. The plants were then wrapped in tin foil and rapidly frozen in liquid nitrogen for 15 minutes. After removal, it was marked separately and placed on dry ice for testing. During the collection of the rhizosphere soil, most of the clay attached to the roots was shaken off, and the remaining soil was gently brushed into a sterile tube and placed on dry ice for testing. Field plant and rhizosphere soil samples were collected from 18 sites. In total, 54 plant and 108 soil samples were tested. Based on the distinction between provinces and regions, the samples from the three sample sites in Xinjiang were marked as A1, A2, and A3, and the samples from the nine sample sites in Inner Mongolia were marked as B1, B2, B3, B4, B5, B6, B7, B8, and B9. Samples from six sample sites in Ningxia were marked as C1, C2, C3, C4, C5, and C6 with three biological repeats in each site.

WheatA wheat germ-agrometeorological big data (V.1.5.6) software (National Centers for Environmental Information [NOAA], https://www.ncei.noaa.gov) was used to extract BIO18 data and latitude and longitude information from the meteorological stations of Xinjiang, Inner Mongolia, Ningxia, and the surrounding areas, including Russia, Mongolia, and Kazakhstan. The climatic layer was processed using the Kriging method during the interpolation analysis. The principle of the Kriging interpolation method is primarily based on the covariance function used to model and predict a random process or field. The results were extracted using a mask according to the desired area.

### 2.3 Transcriptome analysis

RNA was extracted from the root tissue of the samples using the TRIzol method (Yang et al., 2021). An Agilent2100 bioanalyzer (Agilent Technologies, Santa Clara, USA) was used to detect the integrity of RNA. Three biological repeats were performed for each sample site. Using mRNA as a template and random oligonucleotides as primers, the first strand of cDNA was synthesised; the RNA was degraded by RNase H, and the second strand of cDNA was synthesised from dNTPs in a DNA polymerase I system. The purified double-stranded cDNA was repaired by terminal repair, an A-tail was added and connected to the sequencing connector, and the fragment was selected by AMPureXP beads to purify the PCR product. After library construction, different libraries were sequenced using Illumina after pooling according to the effective concentration and target off-machine data demand. The image data obtained using the high-throughput sequencer were transformed into sequence data (reads) using CASAVA base recognition, and the original data were filtered to obtain clean reads. All subsequent analyses were based on high-quality clean reads. First, the sequences were assembled into transcripts using Trinity (v2.6.6), and hierarchical clustering was performed using Corset program transcripts. Using the clustered sequence as a reference, quality control, seven database annotations (NR, NT, KO, SwissProt, PFAM, Gene Ontology (GO), and KOG), quantitative analysis, difference significance analysis, functional enrichment, variation site analysis, and SSR analysis were performed. Weighted gene co-expression network construction (WGCNA), gene expression trend analysis (STEM), and visual analysis of the ipath metabolic pathway were conducted. The screening conditions for differential genes were | log2 (fold change) | > 1, with an error detection rate of < 0.05 (Mao et al., 2005; Kanehisa et al., 2008; Young et al., 2010).

### 2.4 Metabolome analysis

Based on the transcriptome and BIO18 data, representative samples from each production area were selected for wide-target metabolic group detection. The selected samples were A1, A2, B3, B4, B5, C3, and C6. The samples were freeze-dried, removed from the freeze-dryer, and ground to a powder using a grinder (30 Hz for 1.5 min). A 50 mg sample was measured, and 70% methanol-water internal standard extract precooled at 1.2 mL and -20°C was added. Once every 30 minutes, the mixture was vortexed for 30 s, for a total of six times. After high-speed centrifugation (rotating speed of 12000 rpm for 3 min), the supernatant was absorbed and passed through the microporous membrane (0.22 μm) for analysis. The liquid phase conditions were as follows: the mobile phases were A-phase ultra-pure water (0.1% formic acid) and B-phase acetonitrile (0.1% formic acid). The flow rate was 0.35 mL/min, the column temperature was 40°C, and the injection volume was 2 μL. The gradient was 14 min. Analyst 1.6.3 was used to process the mass spectrum data. Based on the local metabolic database, qualitative and quantitative analyses of metabolites were performed using mass spectrometry. The characteristic ions of each substance were screened using a triple quadrupole and the signal strengths (CPS) of the characteristic ions were determined using the detector. The chromatographic peaks were integrated and corrected using Multi Quant software. The statistical function prcomp in R was used for unsupervised principal component analysis (PCA). In both groups, the differences in metabolites with VIP (VIP≥1) and absolute Log_2_FC (|Log_2_FC|≥1.0) were measured. The VIP values were extracted from the OPLS-DA results, which included scores and sorting charts. The R software package MetabAnalystR was used to generate the VIP values. The KEGG database (http://www.kegg.jp/kegg/compound/) was used to identify metabolite annotations by metabolic pathways (http://www.kegg.jp/kegg/pathway.html).

### 2.5 Analysis of rhizosphere soil microbiome

An Ezup column soil Genome DNA extraction kit was used to extract the total DNA of rhizosphere soil microorganisms (Qiu et al., 2023). The universal primers, ITS1 and ITS4, were selected for amplification, and the amplification system and procedure were performed according to the manufacturer’s instructions for the use of high-efficiency and high-fidelity enzymes. Finally, the DNA was purified according to the instructions of the DNA Purification and Recovery Kit. All reagents were obtained from Tiangen Biochemical Technology (Beijing, China). The original data were spliced and filtered to obtain effective data (CleanData). Thereafter, the noise was reduced using DADA2, and sequences with an abundance of less than five were filtered to obtain the final ASVs. Species annotation was performed on the representative sequence of each ASV, and the corresponding species information and abundance distributions were obtained. Simultaneously, the abundance, alpha diversity calculation, Venn map, and petal map of ASVs were analysed to obtain species richness and evenness information and common and unique ASVs information among different samples or groups. However, through the multi-sequence alignment of ASVs and the construction of a phylogenetic tree, we explored the differences in community structure among different samples or groups through dimensionality reduction analysis of PCoA, PCA, non-metric multidimensional scaling (NMDS), and sample cluster tree displays. To further explore the differences in community structure among grouped samples, statistical analysis methods, such as the T-test, MetaStat, and LEfSe, were selected to test the significance of species composition and community structure of grouped samples. The annotation results of the extender were related to the corresponding functional database, and PICRUSt2, BugBase, and Tax4fun software were used to predict and analyse the microbial communities in the ecological samples.

### 2.6 Seedling material and growth conditions

The seeds were collected from Bayannur City, Inner Mongolia Autonomous Region, China (107°03 ‘7.61 “E, 41°05’ 18.73” N). The seeds were soaked in saturated gibberellin solution at 28°C overnight and then soaked in clear water at 28°C overnight to promote seed germination. The treated seeds were cultured in mixed soil with vermiculite:organic soil as 1:3. The temperature of the artificial climate box was maintained at 25°C, the relative humidity at 70%, the light cycle at 12 h day/12 h night, and the average photosynthetically active radiation (PAR) at 152 μmol/m^2^/s. The plants were cultivated for 2 months, water was replenished every day, and growth was observed. Samples were randomly selected for drought treatment and divided into three groups: control (CK), treatment (2 days of drought stress, 5 days of drought stress), and rehydration (watered enough on the 5th day and transferred to the original culture condition for 1 day). After treatment, the seedling samples were quickly retrieved, frozen in liquid nitrogen for 15 min, and placed at -80°C for measurement. The water content of each pot was measured.

### 2.7 Phenotypic observation and soil water content determination of drought stress sample

The plant morphologies of the different groups were observed. The soil water content of each plant was determined using the drying method and the calculation formula was as follows:

Soil water content = (soil weight at sampling time-soil weight after drying)/soil weight at sampling time × 100. The soil moisture content of each group of samples is shown in **Supplementary Table S5**.

### 2.8 Physiological and biochemical index measurements

Plant samples were placed in a homogeniser, and PBS buffer was added, with a plant tissue: homogenate ratio of 1:9. The samples were fully ground under an ice bath for 6 min, transferred to a centrifuge tube, and centrifuged at 12000 rpm for 15 min (4°C). The supernatant was absorbed and divided into 1.5 mL centrifuge tubes and stored at -80°C for testing. An enzyme-linked immunosorbent assay (ELISA) was used to determine the activities of the antioxidant enzymes catalase (CAT), superoxide dismutase (SOD), and peroxidase (POD), as well as the contents of abscisic acid (ABA), Pro, malondialdehyde (MDA) and chlorophyll. R language was used to process and analyse the data. We conducted normality tests (Shapiro–Wilk test) and tests for homogeneity of variance (Levene’s test) on the raw data using the R programming language. Subsequently, we performed statistical analysis and visualisation using one-way ANOVA with GraphPad Prism 8 software. This difference was statistically significant (p < 0.05).

### 2.9 Proteomic analysis

Proteins related to all plant samples under different treatments were determined and analysed by Metware Biotech (Wuhan, China). All plant samples were analysed in triplicate. After the protein was extracted, 8 M urea/100 mM TEAB (pH 8.0) solution was added to re-dissolve the protein. The protein concentration was determined using the bicinchoninic acid method. The samples were stored at -80°C. An appropriate amount of protein was used for 12% SDS-PAGE quality control. Trypsin was used to digest 100 μg protein from each sample. After the protein solution was diluted five times with 100 mM TEAB, trypsin was added at the mass ratio of 1:50 (pancreatic enzyme: protein), and enzymolysis was performed at 37°C overnight. The post-hydrolysis peptide segments were desorbed using a C18 Cartridge and freeze-dried. The peptide lyophilised powder was redissolved in 0.1% formic acid water at 0.1 µg/µL and stored at -20°Cfor later use. The extracted protein was analysed using liquid chromatography-mass spectrometry (LC-MS)/MS. After chromatographic separation, the sample was collected using the PASEF mode of timsTOF Pro mass spectrometer. The data were analysed and processed using FragPipe (v17.1) software. According to the ncbi_Lithospermeae_zicao_20220812. fasta database, anti-decoys and contaminants were added to control the false-positive rate (FDR) from random matches and eliminate the effects of contaminating proteins. Label-free quantification was performed using the MaxLFQ algorithm of the IonQuant module. Comprehensive functional annotation of the identified proteins and differentially expressed proteins in each comparison group was performed using GO, KOG functional classification, KEGG pathway, protein domain, subcellular localisation, and SignalP. In addition, R software (clusterProfiler) (v3.10.1) was used to conduct enrichment analysis of the differentially expressed proteins in each comparison group at four levels: GO classification, KOG functional classification, KEGG pathway, and protein domain, to comprehensively determine the physiological functions of the differentially expressed proteins.

### 2.10 PRM verification

To verify the reliability of the detection of specific proteins, we selected nine proteins for PRM quantitative detection. Proteins were extracted from the sample and quantitative PRM analysis was performed on the target peptide after enzyme digestion. The peptide information suitable for PRM analysis was imported into Xcalibur software for the PRM method setting. Approximately 1 µg peptide was taken from each sample and mixed with 20 fmol standard peptide (PRTC: ELGQSGVDTYLQTK) for detection. This test was conducted by Zhongke New Life Biotech (Shanghai, China). The target peptides of nine target protein types were analysed using Skyline software. Based on the relative expression of the corresponding peptide of each target protein in the different sample groups, the relative difference in the expression of the target protein was calculated.

### 2.11 qRT-PCR validation

qRT-PCR was used to verify the proteins involved in shikonin biosynthesis and the randomly selected differential proteins. Thirteen protein-related genes were selected and detailed information is provided in **Supplementary Table S6**. Total RNA was extracted from each group and three biological replicates were obtained for each group. After the RNA was reverse transcribed into cDNA, the amplification system was formulated using the TB Green Premix Ex Taq II kit. The amplification system consisted of 10 µL TB Green Premix Ex Taq II, 0.4 µL ROX Reference Dye (50X), 0.8 µL 10 µmol/L upstream primer, and 0.8 µL 10 µmol/L downstream primer. Two-microliter cDNA template was composed of 6 µL double distilled water for a total volume of 20 µL. The amplification procedure was 95°C for 30 s (pre-incubation), 95°C for 5 s, and 60°C for 34 s, for a total of 40 cycles (two-step amplification); and 95°C for 5 s, 60°C for 60 s, and 95°C for 15 s (dissolution). ACT7 was used as the internal reference gene, and the relative quantitative analysis of the data was calculated using the 2^−ΔΔCT^ method.

### 2.12 Metabolomic analysis

The contents and species of metabolites in the different groups were analysed based on the results of widely targeted metabolomics data determined by Metware Biotech (Wuhan, China). All plant samples were analysed in triplicate. The samples were vacuum-dried in a freeze-dryer for 2 d, ground with a grinder (30 Hz, 1.5 min) to a powder form, and dissolved in 1.2 mL of 70% methanol solution. Analyst 1.6.3 was used to process the mass spectrum data. Based on the metware database (MWDB), the secondary spectral information was used to characterise the materials based on the MWDB. The KEGG database (http://www.kegg.jp/kegg/compound/) was used to identify the metabolite annotations and annotation of metabolites by metabolic pathways in the KEGG database (http://www.kegg.jp/kegg/pathway.html).

### 2.13 Prediction of future adaptive regionalisation of *A. guttata*

To reduce the influence of the correlation of environmental factors on the model, Pearson was used to test the multicollinearity between environmental factors. Fifteen variables, including seven meteorological data and eight soil data under current climatic conditions, were selected for modelling to predict the suitable distribution area of *A. guttata* in the future. Future meteorological data were obtained from the World Climate Database (http://www.worldclim.org). Soil data were derived from the Spatial Information Grid Database of Traditional Chinese Medicine Resources (http://www.tcm-resources.com/). The spatial resolution of the data was 30 arcsec. Maxent and ArcGIS were used to generate the pictures.

The MaxEnt settings are as follows: the MaxEnt output mode was “Logisitc”, the maximum number of iterations of the model was 105, the convergence threshold was 0.0005, and other parameters were defaulted to 10 times cross-validation. To ensure the accuracy of the model prediction, the AUC was used to evaluate the model’s prediction results. An AUC value greater than 0.75 indicates a relatively good prediction ability of the model.

## 3 Results

### 3.1 *A. guttata*: history, resources, and national zoning

*A. guttata* tastes sweet, salty, and cold and returns to the heart and liver meridians. It has the effects of clearing heat and cooling the blood, promoting blood circulation and detoxification, penetrating rashes, and eliminating spots. It is comprised of perennial herbs with yellow corollas and pubescence. According to the 2020 edition of the Chinese Pharmacopoeia, the main source of Arnebiae Radix is the dried root of *Arnebia euchroma* or *A. guttata*. Its active components include naphthoquinone compound L-shikonin, β, and β’- dimethylacryloyl aconine.

Arnebiae Radix as a medicinal material was first reported in “Shen Nong’s herbal classic”, “Zicao Jie Chi, February flower … Born in Dangshan and Chu … can be dyed purple.”, but *A. guttata* as a medicinal herb was probably recorded in the Western Jin Dynasty. In the “Natural History” of Zhang Hua in the Western Jin Dynasty (232–300), “Pingshi Yangshan Arnebiae Radix is very good”, Pingshi Yangshan refers to Langshan Mountain in Inner Mongolia, which is located in Bayannur City. In 1964, Xie Zongwan listed *A. guttata* as one of the four medicinal bases of Arnebiae Radix in the “Discussion on Varieties of Traditional Chinese Medicine”. In Inner Mongolia Chinese Herbal Medicine published in 1972, “*A. guttata …* Corolla bright yellow”, has been determined to be *A. guttata.* In a resource survey conducted in the 1870s, Academician Xiao Peigen confirmed that *A. guttata* was widely used locally as a commodity of Arnebiae Radix and included it in the “New Chinese Medicine Records” that was published in 2002. In 1990, *A. guttata* was officially incorporated into the Pharmacopoeia, which may have been due to the high demand for *A. euchroma*; however, it is wild and overexploited, resulting in a shortage of resources and its listing as a national second-class endangered plant. However, *A. guttata* and *A. euchroma* exhibit similar genetic relationships, sufficient yields, and definite curative effects. Currently, *A. guttata* is a wild resource and artificial planting technology is not mature; therefore, the exploration of planting methods and resource development is of great relevance.

**Figure 1:**
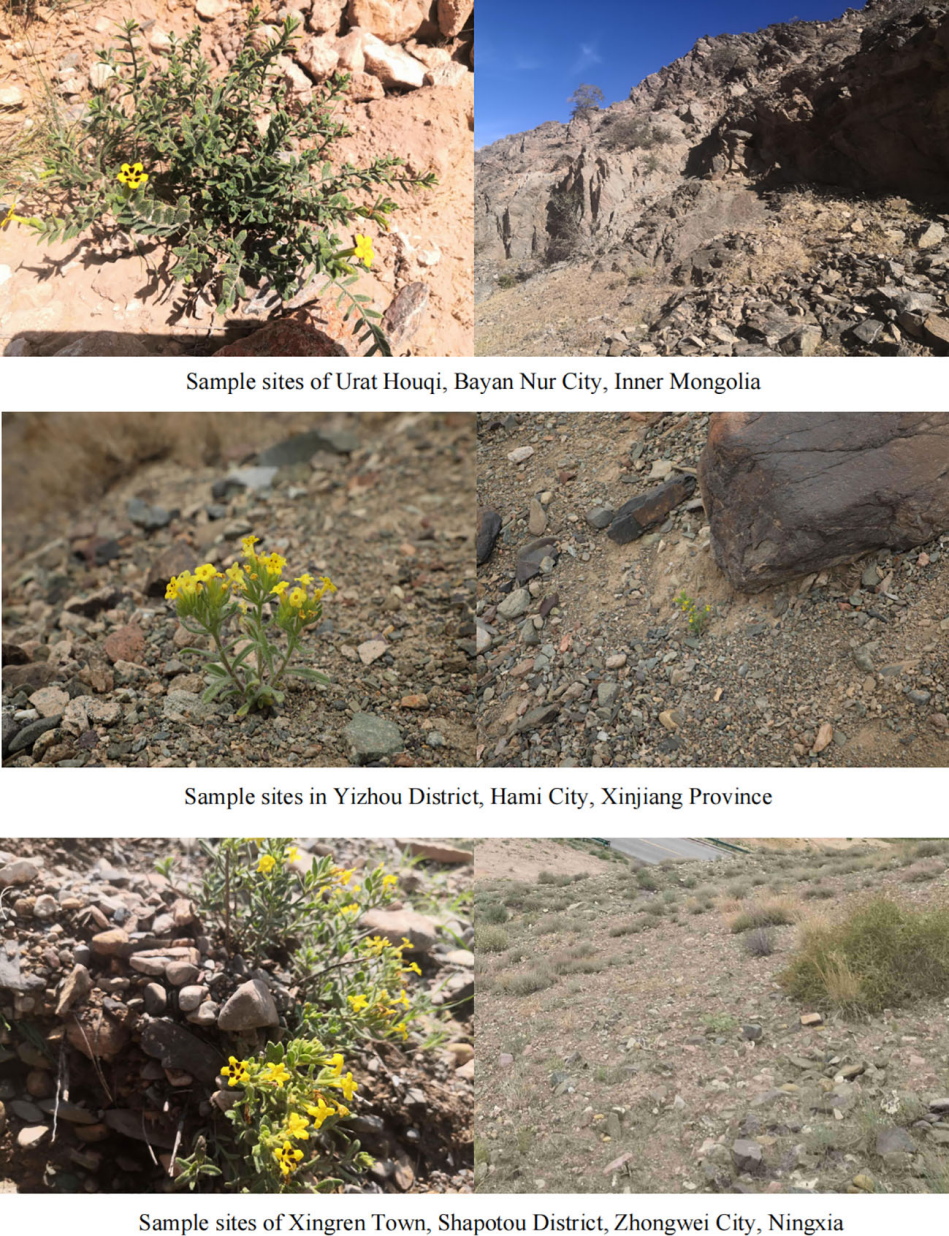
Habitats of different *A. guttata* cultivation areas.

According to the previous resource survey records, *A. guttata* primarily grows in deserts, sand, gobi, lakeside stones, and sunny gravel hillsides. Through field investigations and visits, it was found that the output was abundant in Inner Mongolia and Ningxia, a small amount was distributed in Xinjiang, and there was no distribution in the visited areas of Gansu. The MaxEnt model’s relative contribution indicates that BIO18 was the largest contributing factor, followed by altitude (**Table 1**). Specific information on the samples is presented in **Supplementary Table S1**; **Figure 1** shows the habitat of *A. guttata.*

**Table 1:** Contribution of ecological factors to growth suitability zoning of *A. guttata*.

Ecological factor	Percent contribution (%)
BIO18	21.2
Elevation	19.2
pH value	16.3
BIO4	14.5
BIO2	6.9
BIO9	5.7
Soil effective moisture content level	5.2
BIO15	4.9
BIO19	1.8
Soil type	1.1
Organic carbon content	0.9
Soil texture classification	0.8
BIO1	0.7
Soil cation exchange capacity	0.4
Clay content	0.3

The sample site with the highest BIO18 value was Ningxia, followed by Inner Mongolia and Xinjiang. The BIO18 value of Xinjiang was significantly different from that of the other two provinces and regions, which is only approximately 1/20–1/5 of the other two provinces’ BIO18. The BIO18 values also varied across provinces. For example, in Inner Mongolia, the BIO18 value in the Yinshan area was the lowest, and the BIO18 value in Bayinbaolige Town, Wulat Hou Banner, was in the middle of all sample points. The distribution of 18 sample points for the reference BIO18 is illustrated in **Supplementary Figure S1**. The BIO18 values for each sampling point are listed in **Supplementary Table S2**.

### 3.2 Significant genetic differences among *A. guttata* samples from different production areas

A total of 718574 genes were identified in the 54 samples. The correlation between gene expression levels among the samples was evaluated. From the correlation analysis, we can observe that *A. guttata* from different regions in the wild were unlikely to exhibit global differential expression in RNA-seq. Instead, there were probably differences in the expression of certain specific genes. Additionally, the PCA showed similar results (**Supplementary Figure S2**).

**Figure 2:**
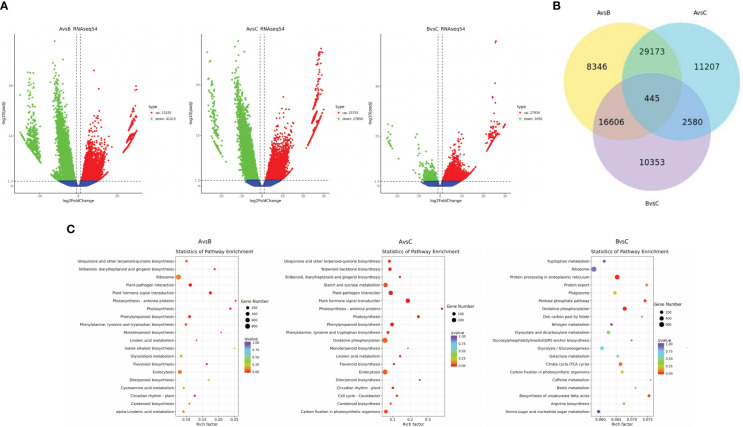
Differential gene volcano map **(A)**. Venn diagram of differential genes **(B)**. KEGG path rich and scattered point graph **(C)**.

Considering the production area as a large group, the differentially expressed genes (DEGs) were analysed (**Figure 2A**), and the transcript differences among the samples from different production areas were explored. In the A vs. B group, 13155 DEGs were upregulated and 41415 DEGs were downregulated. In the A vs. C group, 15755 DEGs were upregulated and 27650 DEGs were downregulated. In the B vs. C group, 27934 DEGs were upregulated and 2050 DEGs were downregulated. There were significant differences in gene expression among samples from different production areas.

The common DEGs among the comparison groups were analysed, and DEGs with special biological significance were screened (**Figure 2B**). Groups A vs. B and A vs. C contained the highest number of DEGs (29,618). A total of 445 DEGs were identified in the three comparison groups. There were 54570 DEGs between groups A and B, 43405 DEGs between groups A and C, and 29984 DEGs between groups B and C. There are more significant differences in gene expression among groups A, B, and C.

**Figure 3:**
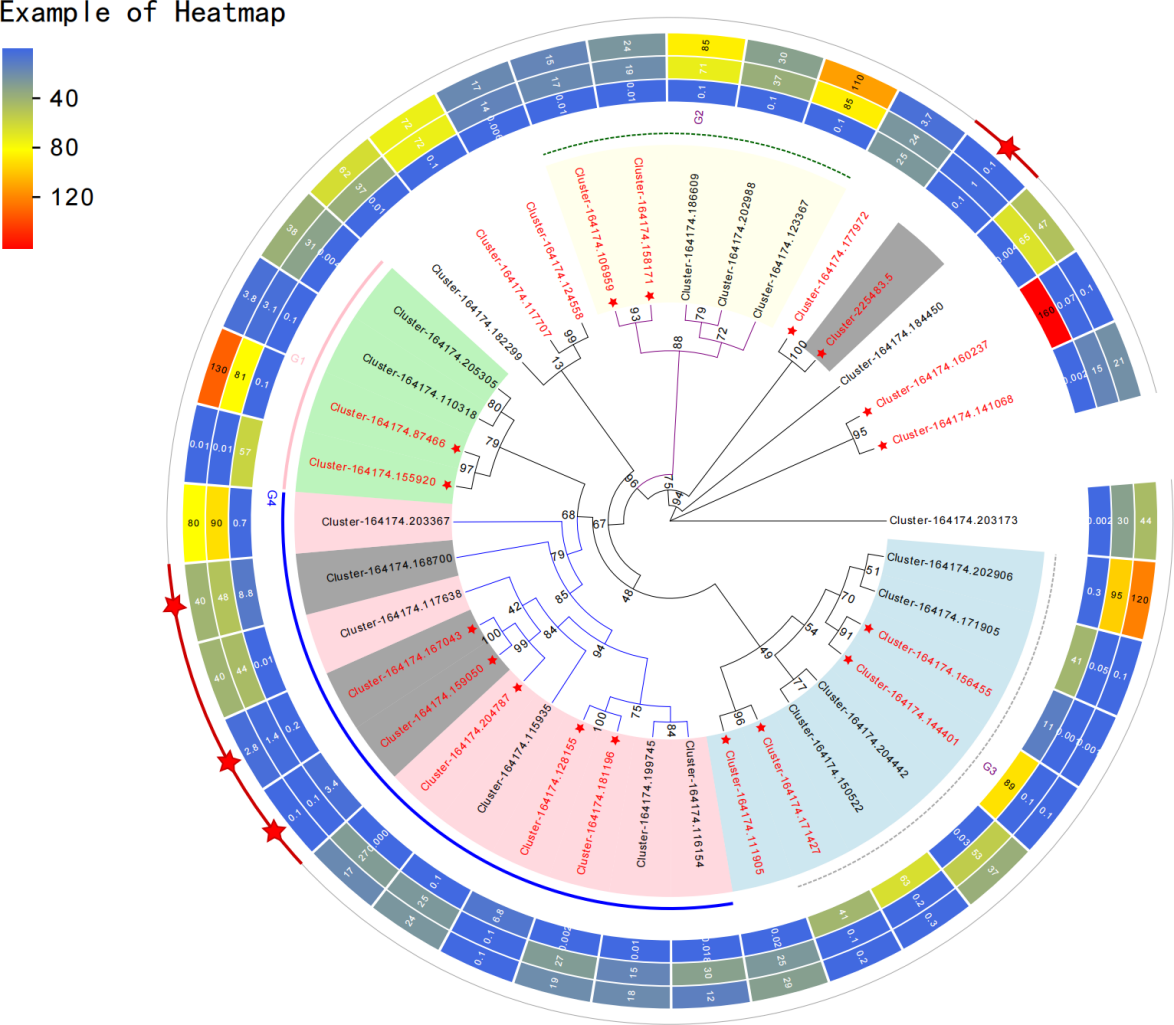
Genetic evolution analysis of representative differentially expressed genes. The heat map shows the expression of each gene in the samples of the three production areas.

The KEGG clustering patterns of DEGs among groups were explored, and differences in gene expression and regulatory pathways among different appellation areas were analysed (**Figure 2C**). DEGs of groups A and B were mainly clustered in plant hormone signal transduction (ko04075), phenylpropanoid biosynthesis (ko00940), ubiquinone and other terpenoid-quinone biosynthesis (ko00130), flavonoid biosynthesis (ko00941), circadian rhythm plant (ko04712), photosynthesis (ko00195), etc. DEGs of groups A and C were mainly enriched in plant hormone signal transduction (ko04075), oxidative phosphorylation (ko00190), phenylpropanoid biosynthesis (ko00940), starch and sucrose metabolism (ko00500), ubiquinone and other terpenoid-quinone biosynthesis (ko00130), terpenoid backbone biosynthesis (ko00900), flavonoid biosynthesis (ko00941), circadian rhythm plant (ko04712), carotenoid biosynthesis (ko00906), carbon fixation in photosynthetic organisms (ko00710), etc. DEGs in groups B and C were mainly concentrated in the biosynthesis of unsaturated fatty acids (ko01040), protein processing in the endoplasmic reticulum (ko04141), pentose phosphate pathway (ko00030), citrate cycle (ko00020), etc. The main regulatory pathways of DEGs enrichment between groups A and B and between groups A and C were similar and more concentrated in the process of secondary metabolism, especially in the enrichment of ubiquinone and other terpenoid-quinone biosynthesis. This pathway regulates shikonin biosynthesis. The DEGs between groups B and C mainly regulated the primary metabolic processes of the substances. Among the genes of the three origin samples, 37 representative genes (CDS) with significant differences owing to different regions and climates were selected for genetic evolution analysis (**Figure 3**). The results showed that the selected genes could be divided into four main branches, represented by light green, light yellow, light blue and light pink (G1-G4). Among them, Cluster-164174.141068 and defensin SD2 (Cluster-164174.160237), transaldolase (Cluster-164174.177972) and cytochrome P450 monooxygenase (Cluster-225483.5), B2 protein (Cluster-164174.158171) and albumin-1 (Cluster-164174.106959), ERF transcription factor (Cluster-164174.124558), and transcription factor BHLH148-like (Cluster-164174.117707) genes are closely related or homologous genes. *A. guttata* contains shikonin and phenolic acid and other active ingredients. In the screened genes, ACC oxidase 1, 4-coumarate: CoA ligase (Cluster-164174.168700) and cytochrome P450 monooxygenase psoD-like are important for regulating the synthesis of these two components. Among transcription factors, the ERF and bHLH families play key roles in plant resistance to drought.

### 3.3 Significant metabolite differences among *A. guttata* samples from different production areas

**Figure 4:**
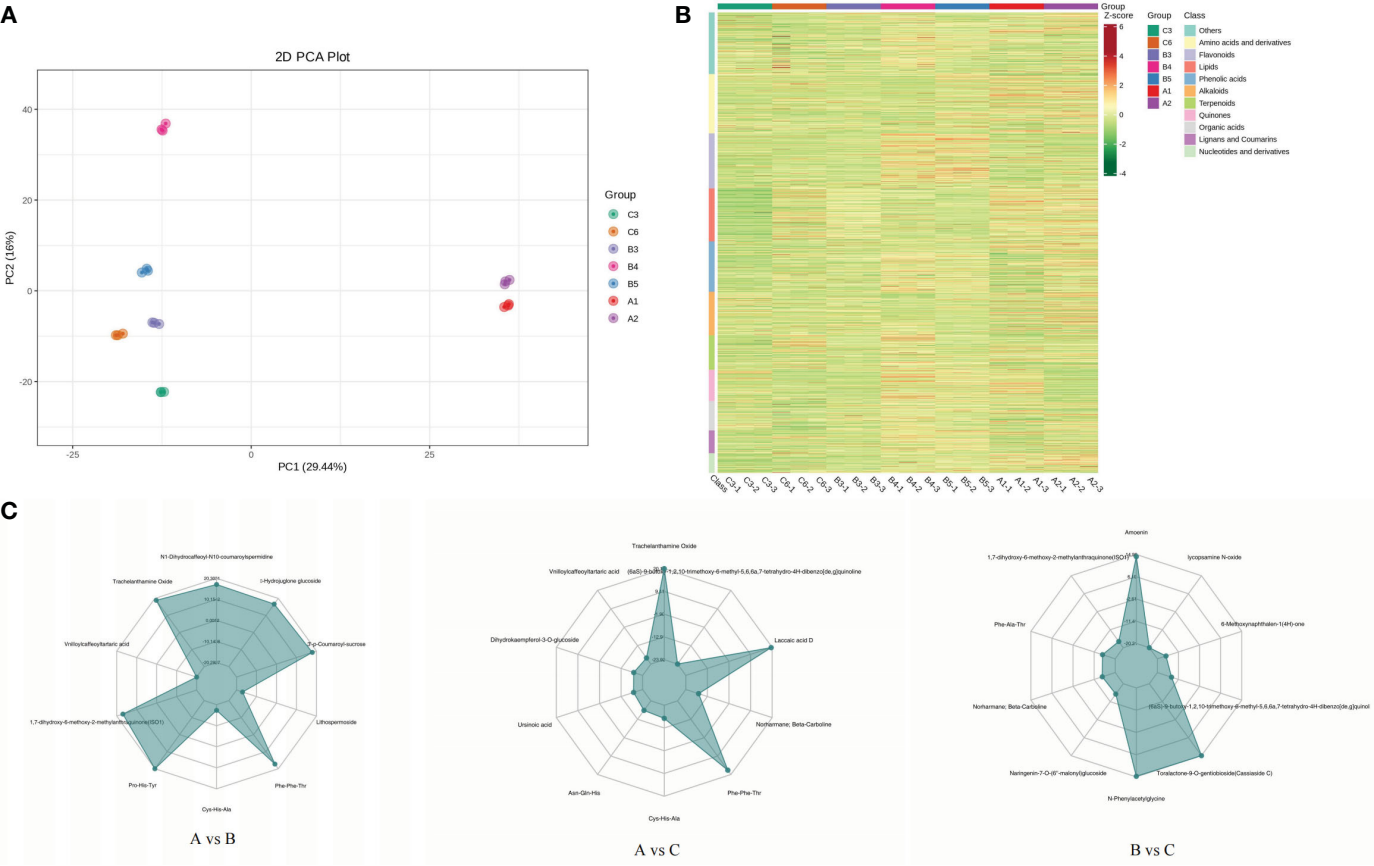
PCA score chart of each group of samples **(A)**. Overall sample cluster diagram **(B)**. Radar map of differential metabolites **(C)**.

The PCA results showed significant differences in the PC1 and PC2 levels among the different samples, with small intragroup differences and good repeatability (**Figure 4A**). The results showed a high degree of separation between group A and the other two groups, indicating significant differences in the formation and accumulation of metabolites. All samples and metabolites were clustered, and the macroscopic relationships between the total samples and metabolites were analysed (**Figure 4B**). As shown on the cluster heat map, the metabolites of the two sample points in group A were clustered into one group. The distribution of metabolites in group B was also similar, and there was a certain relationship between the distribution of C6 metabolites and group B metabolites. The samples from each group were further analysed by hierarchical cluster analysis to form a cluster tree. The samples of groups B and C were clustered into one branch, and the samples of group A were clustered into another. In group B, the samples of groups B3 and B4 were clustered into one branch. Compared with group C, B3, C3, and C6 were clustered together, and B4 and B5 were clustered into one group (**Supplementary Figure S3**). The metabolite accumulation patterns of the samples that aggregated into one branch were similar. Analysis of the differences in metabolite content among the different groups is of great significance for studying the material formation process of samples from different production areas (**Figure 4C**). The metabolites with significant differences between groups A and B included N1-dihydrocaffeoyl-N10-coumaroylspermidine (alkaloids), α-hydrojuglone glucoside (quinones), 3’-p-coumaroyl-sucrose (phenolic acids) lithospermoside (alkaloids), Phe-Phe-Thr (amino acids and their derivatives), 1,7-dihydroxy-6-methoxy-2-methylanthraquinone (anthraquinones), vinilloylcaffeoyltartaric acid (phenolic acid), and trachelanthamine Oxide (pyrrole alkaloid). Laccaic acid D (anthraquinone), Asn-Gln-His (amino acid and its derivatives), dihydrokaempferol-3-O-glucoside (dihydroflavonol), and vinilloylcaffeoyltartaric acid (phenolic acids) were significantly different between groups A and C. There were significant differences in metabolites between groups B and C, including Amoenin (flavonol), lycopsamine N-oxide (pyrrole alkaloids), 6-methoxynaphthalen-1(4H)-one (quinones), toralactone-9-O-gentiobioside (naphthols), N-phenylacetylglycine (amino acids and their derivatives), and naringenin-7-O-(6’’-malonyl) glucoside (dihydroflavonoids).

Shikonin (including the isomer aganin) is a unique and main medicinal component in *A. guttata*, and its content is an important basis for evaluating the quality of *A. guttata*. Therefore, we analysed the content of shikonin to analyse the accumulation of effective components in samples from different production areas. According to the qualitative and quantitative results, 17 shikonins were identified. The sum of all shikonin contents was calculated, and the highest were A1 (1.51E+08) and B5 (1.22E+08). The BIO18 value of the A1 point was relatively low (30.285 mm), whereas the BIO18 of the B5 point was in the median of all sampling points, and the precipitation in the warmest season was moderate. The shikonin content of C6 was the lowest. The BIO18 value was 200.422 mm, which was the highest at all sample points.

### 3.4 Significant soil microbial community differences among *A. guttata* samples from different production areas

**Figure 5:**
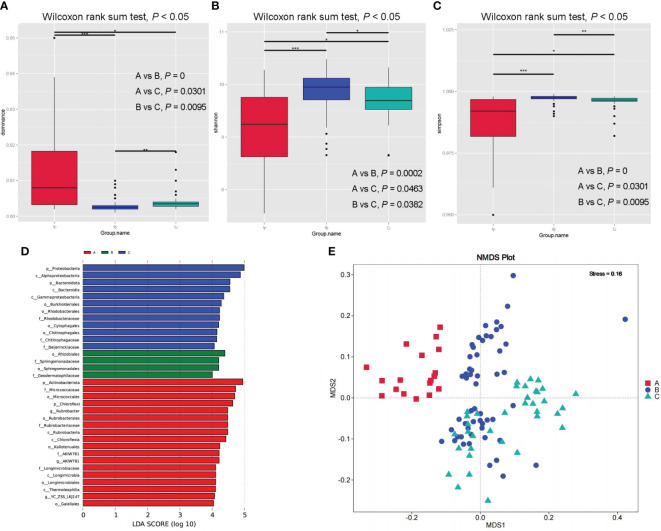
Rhizosphere bacterial communities of samples from different producing areas box plot of dominance index of species uniformity **(A)** and box plot of Shannon species diversity index **(B)** and box plot of Simpson species distribution diversity and evenness index differences **(C)**. Evolutionary cladogram of rhizosphere bacterial communities from different producing areas **(D)**. Differences of bacterial community composition in rhizosphere of samples from different producing areas based on NMDS **(E)**.

After paired end-to-end alignment, mass filtration, chimerism, and deletion of a single gene, mitochondrial, and chloroplast sequences, a total of 48244 operational taxonomic units (OTUs) were found in all samples. In general, the number of OTUs gradually decreased from Inner Mongolia to Ningxia and then to Xinjiang, with significant differences between Xinjiang and Inner Mongolia (pA-vs-B = 0.025, p < 0.05); however, there were no significant differences between Inner Mongolia and Ningxia and Xinjiang and Ningxia (pB-vs-C = 0.394, pA-vs-C = 0.136, p > 0.05). The total number of OTUs in the three major production areas was 4099. The number of OTUs shared by Inner Mongolia and Ningxia was the largest (10660), whereas that shared by Xinjiang and Ningxia was the lowest (4861). Alpha diversity revealed significant differences in the rhizosphere bacterial community diversity among samples from Xinjiang, Inner Mongolia, and Ningxia. Community species diversity in Inner Mongolia and Ningxia was significantly higher than that in Xinjiang, whereas that in Inner Mongolia was slightly higher than that in Ningxia (**Figures 5B, C**). In addition, the dominant species evenness index calculated using the Wilcoxon rank-sum test showed that the species evenness in Xinjiang was significantly higher than that in Inner Mongolia and Ningxia and that in Ningxia was slightly higher than that in Inner Mongolia (**Figure 5A**). NMDS and anosim analyses based on unweighted UniFrac distances showed significant differences in the rhizosphere bacterial community and composition among samples from different regions (stress = 0.16, stress < 0.2; R = 0.4174, p = 0.005), indicating that each cultivation area and soil had a distinct population (**Figure 5E**; **Supplementary Table S3**).

A total of 61 phyla, 164 classes, 405 orders, 680 families, and 1525 genera were detected by analysing the composition of the rhizosphere bacteria. The top 10 dominant bacteria in the rhizospheres of the samples from the three production areas were the same; however, there were significant differences in the abundance of bacteria in the different production areas and at different levels. Therefore, based on the species abundance at different levels, MetaStat was used to screen for species with significant differences between the groups. The results showed significant differences in the biomarkers among the three production areas (**Supplementary Table S4**). Simultaneously, the LDA Effect Size (LEfSe) method was used to identify the differentially enriched bacterial communities among the rhizosphere soils of the samples from the three major production areas (**Figure 5D**; **Supplementary Figure S4**). By combining the LEfSe and MetaStat methods, 16 bacterial biomarkers with significant differences at different classification levels were identified in rhizosphere soil samples from Xinjiang, Inner Mongolia, and Ningxia. These included Actinobacteria, Proteobacteria, Chloroflexi, Bacteroidetes, Bacteroidia, Rubrobacteria, Chloroflexia, Micrococcales, Rhodobacterales, Rubrobacterales, Gaiellales, Micrococcaceae, Rhodobacteraceae, Rubrobacteriaceae, Longimicrobiaceae, and Rubrobacter.

### 3.5 Association between genes, metabolites, and soil microbial communities in field *A. guttata* samples

In each comparison group, the expression of trans-cinnamic acid 4-monooxygenase and 4-coumarate-coenzyme A ligase, the key regulatory genes of shikonin biosynthesis, was consistent with the trend of shikonin accumulation. Trans-cinnamic acid, the key substance in shikonin biosynthesis, was similarly expressed. Other genes may also have regulated shikonin synthesis. For example, Cluster-164174.132198 can regulate the transcription of DNA templates, and negatively regulate the synthesis of angeloylshikonin and β, β-dimethylacrylyl shikonin. Serine-protein kinases ATM and isobutyl shikonin were also negatively regulated. The cohesin complex subunit SCC1, transcription factor SPT5, B-cell receptor-associated protein 31, and long-chain acyl-CoA synthetase negatively regulated deoxyshikonin synthesis.

A correlation between key metabolites and microorganisms was found. A strong negative correlation was observed between Chloroflexia and acetylacanin. Rubrobacteria, Rubrobacteriaceae, *Rubrobacter*, Rubrobacterales and Actinobacteriota were negatively correlated with the synthesis of acetylshikonin, alkannin, and shikonin. The correlation between metabolites and soil microorganisms indicated that the metabolism of *A. guttata* was affected by the composition and activity of the microorganisms.

In summary, we found significant differences and correlations in gene expression, material accumulation, and soil microbial community composition among samples from different habitats. There was also a relationship between the maximum contribution factor BIO18 and shikonin content in the sample. BIO18 may have affected the synthesis and accumulation of shikonin. Precipitation controls the water content of the soil, and insufficient water leads to drought stress. This study showed that in Inner Mongolia, Xinjiang, Ningxia, and other northwestern regions, drought is the most important natural factor restricting plant and ecological development (Chen et al., 2023). Therefore, in the following sections, the metabolic process of *A. guttata* under drought stress is discussed.

### 3.6 Prediction of suitable growth areas of *A. guttata* in the next 40 years

Using MaxEnt software, the contribution rates of various ecological factors affecting the growth of *A. guttata* were obtained, and the climatic factor with the highest contribution was still BIO18 (**Table 2**).

**Table 2:** Contribution of ecological factors to future growth suitability zoning and distribution of *A. guttata*.

Ecological factor	Percent contribution (%)
BIO18	43.2
pH value	19.6
BIO4	10.3
BIO2	6.9
Soil effective moisture content level	5.9
BIO1	3.4
BIO9	3
BIO15	2.9
BIO19	2.4
Soil cation exchange capacity	1.2
Organic carbon content	0.5
Soil texture classification	0.4
Soil type	0.3
Clay content	0

The potentially suitable distribution areas for *A. guttata* under future climates of two different periods were predicted, and the currently suitable distribution areas were compared. The results show that under current climatic conditions, the most suitable areas are mainly located in northern Xinjiang, northern Gansu, central and western Inner Mongolia, and a few areas in northern Ningxia. The most suitable growth area showed a radiating trend.

**Figure 6:**
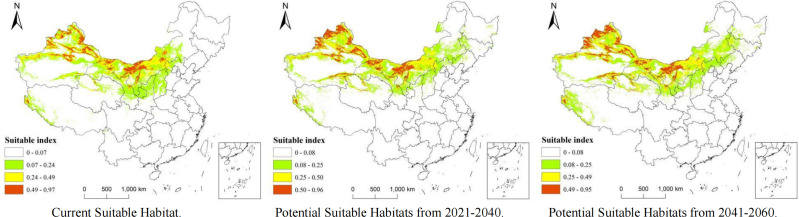
Current and projected suitable habitats for A. guttata from 2021–2060 based on MaxEnt.

Compared with the current climatic conditions, the most suitable distribution area for *A. guttata* in the 2021–2040 and 2041–2060 periods showed a general westward trend, and the distribution in northwestern and southern Xinjiang and northern Gansu was significantly higher than that under the current climatic conditions (**Figure 6**). In Inner Mongolia, the optimal distribution areas for *A. guttata* in the Xilin Gol League, while Ulanqab City showed a decreasing trend. The trend of the more suitable distribution areas is similar to that of the most suitable distribution areas; however, the distribution of the more suitable distribution areas will increase in eastern Mongolia over the next 40 years.

### 3.7 Phenotypic characteristics of *A. guttata* under drought stress

**Figure 7:**
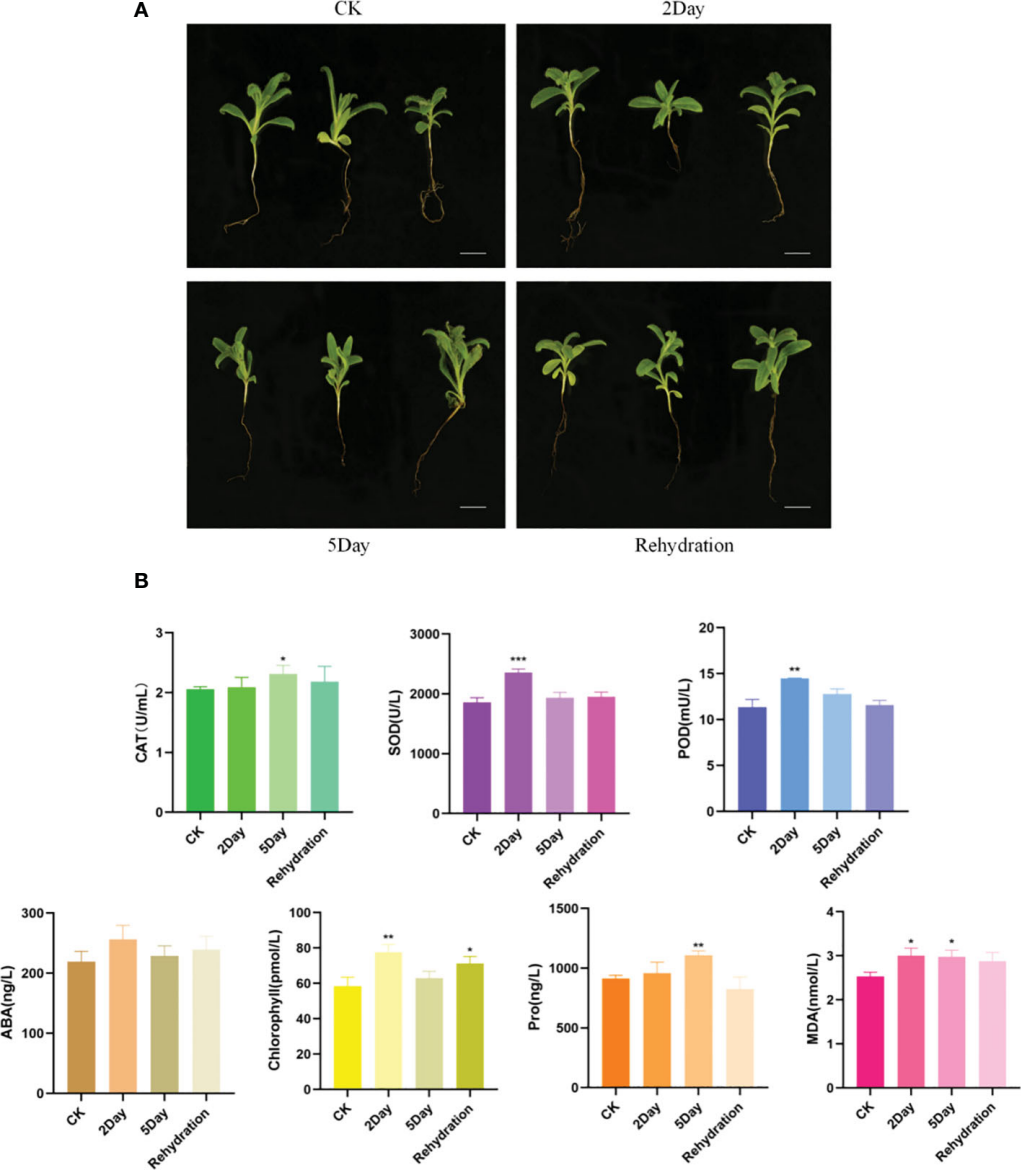
Phenotypic morphological changes of *A*. *guttata* **(A)** and physiological and biochemical indexes **(B)**. Scale bar, 2 cm. (n = 3. *p < 0.05; **p < 0.01; ***p < 0.001).

Morphological observations showed that seedling samples did not change significantly during the 2Day drought stress, but in the 5Day drought stress, leaves began to straighten and converge and underwent leaf thinning, followed by wilting. After rehydration, plant leaves began to stretch, fill, and thicken, and were essentially restored to the state before drought stress (**Figure 7A**). This indicated that *A. guttata* could resist mild drought and maintain normal growth; however, severe drought affected its life activities with various phenotypic effects. Seedlings returned to normal after rehydration.

### 3.8 Significant changes in physiological indexes in the drought response

Drought stress affected the physiological indices of *A. guttata* seedlings (**Figure 7B**). The activity of SOD, POD, and CAT increased significantly under drought stress and reached the peak during the 2Day drought or 5Day drought. When the seedlings were rehydrated, enzyme activity tended to be stable, indicating that it played an important role in drought resistance. Under drought stress, the content of ABA in seedlings increased to a certain extent, reached the highest level during the 2Day drought stress, and then decreased during the 5Day drought stress. After rehydration, the ABA content was almost the same as that in the CK group, achieving drought resistance. Chlorophyll content increased first and then decreased, and the content was the highest in the 2Day drought. With increasing drought stress, the Pro content in seedlings gradually increased, and it was most significant in the 5Day drought stress treatment. The results showed that the content of MDA in seedlings increased under drought stress, whereas the content of MDA in the 2Day and 5Day droughts was basically the same, which was not significantly higher than that in the CK. The MDA content did not increase significantly even when the stress level increased, indicating a strong ability of *A. guttata* to withstand drought stress.

### 3.9 Drought stress significantly affects metabolic processes

**Figure 8:**
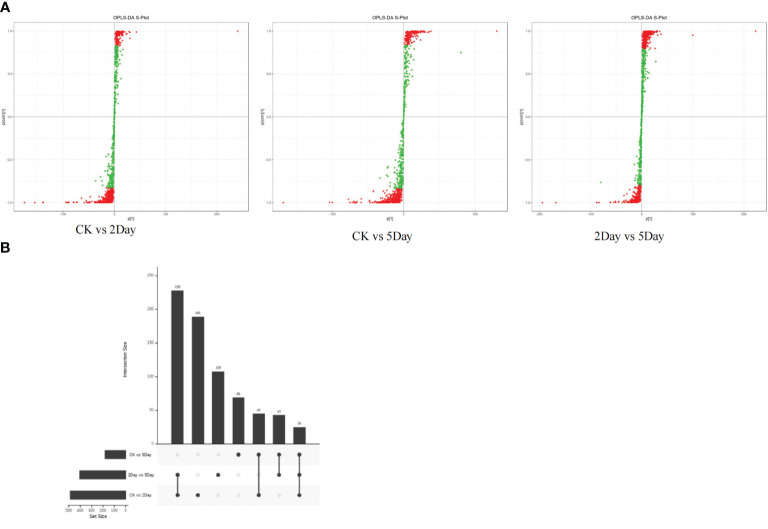
OPLS-DA S-plot **(A)**, and DEPs upset diagram **(B)**.

UPLC-MS/MS and 4D-lable-free quantitative analysis techniques were used to analyse the metabolic changes in *A. guttata* during drought. The results showed that the four groups of samples under different drought stress conditions had significant differences in metabolites, and the related regulatory proteins showed the same content trend (**Supplementary Figure S5**). The concentrations of quinones, phenolic acids, alkaloids, flavonoids, terpenoids, carbohydrates, amino acids, and their derivatives differed significantly under drought stress conditions (**Figure 8A**). All identified proteins were screened for significant differences, and the protein expression patterns among the treatment and comparison groups were analysed. There were 25 differentially expressed proteins (DEPs) common among the three comparison groups (**Figure 8B**). In the CK vs. 2Day comparison groups, there were 487 DEPs, of which 41 were upregulated and 446 were downregulated. In the CK vs. 5Day comparison groups, there were 182 DEPs, of which 49 were upregulated and 133 were downregulated. In the 2Day vs. 5Day comparison groups, there were 404 DEPs, of which 330 were upregulated and 74 were downregulated (**Supplementary Figure S5**).

**Figure 9:**
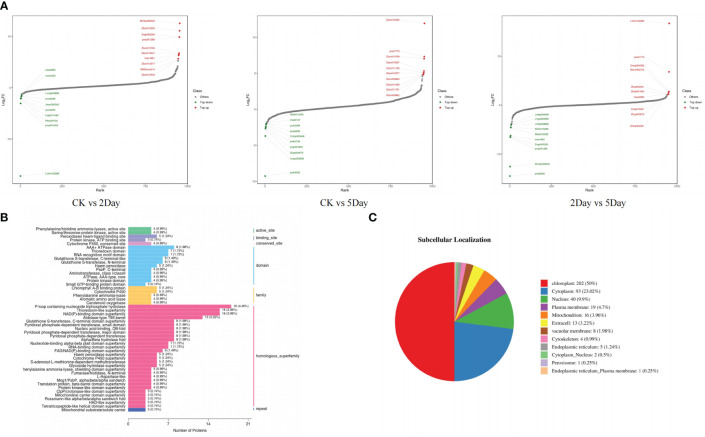
Dynamic distribution map of metabolite content difference **(A)**, domain annotation bar chart **(B)**, and pie chart of subcellular localisation results **(C)**.

In the CK vs. 2Day groups, shikonin (including isoamyl shikonin and propanyl shikonin), quinones (including 1/4-methoxylithospermidin A and 1/4-methoxylithospermidin B), flavonoids (including geranyin, Acacia-7-O-rutin) and other metabolites were most significantly upregulated. In the CK vs. 5Day groups, shikonin (including shikonin, acetyl shikonin, and acetylakanin), quinones (including 1/4 methoxylithospermidin A and 1/4 methoxylithospermidin B), flavonoids (including centathrin-3-O-rutin) and other metabolites were the most significantly upregulated. In the 2Day vs. 5Day groups, flavonoids (including centaurin-3-O-rutin and quercetin-3-O-(6”-o-acetyl)-glucoside), alkaloids (including intermedine and rinderine), phenolic acids (including 2-O-cafeoylmalic acid and coniferol) and other metabolites were the most significantly upregulated. The results showed significant changes in the types and contents of metabolites under different drought stress levels (**Figure 9A**).

**Figure 10:**
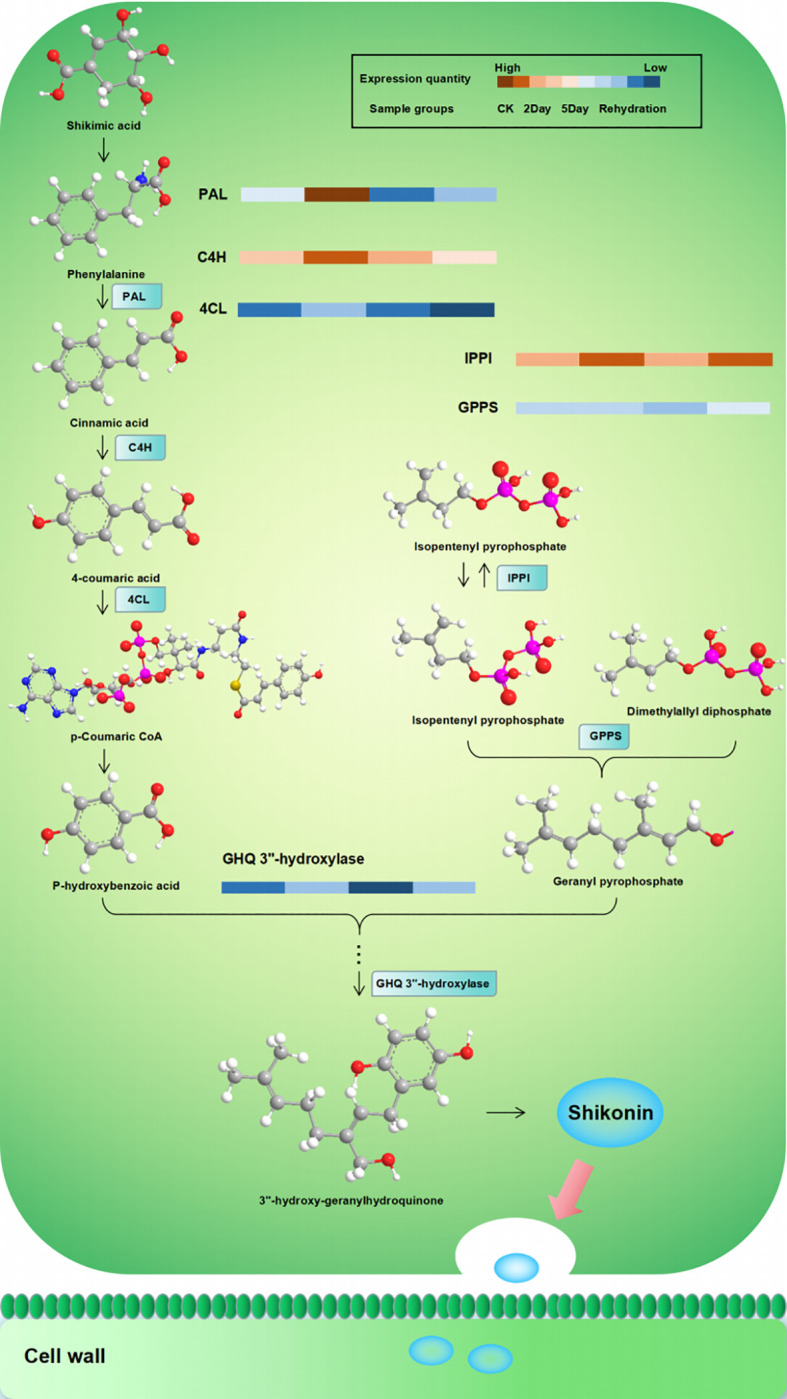
Regulation of enzymes related to the biosynthesis of shikonin under drought stress. PAL, phenylalanine ammonia-lyase; C4H, cinnamic acid 4-hydroxylase; 4CL, 4-coumarate:CoA ligase; IPPI, isopentenyl pyrophosphate:dimethyllallyl pyrophosphate isomerase; GPPS, geranyl diphosphate synthase; GHQ 3”-hydroxylase, geranylhydroquinone 3’’-hydroxylase.

DEPs annotated by all secondary GO items under the three primary classifications (molecular function (MF), biological process (BP), and cellular component (CC)) were counted. As shown in **Figure 10**, in the CK vs. 2Day comparison groups, the main functions of DEPs under BP were annotated as metabolic process, cellular process, regulation of biological process, response to stimulus, and others. Most DEPs were downregulated, whereas the upregulated DEPs included 4-coumarate:coenzyme A ligase, phenylalanine ammonia lyase, cinnamic acid-4-hydroxylase, dimethylallyl pyrophosphate isomerase, hydroquinone-3”- hydroxylase, and others. The main functions of DEPs in the CC category were described as cell parts, membrane parts, and protein-containing complexes. The upregulated DEPs included KAG9160943.1, KAG9160943.1 and the other predicted proteins. These proteins are involved in shikonin biosynthesis (Auber et al., 2020). The main functions of DEPs in the MF category were catalytic activity, structural molecule activity, and binding. The upregulated DEPs included 4-coumaric acid:CoA ligase 3, KAG9138459.1, KAG9138459.1, and others. In the 2Day vs. 5Day comparison groups, DEPs in the BP category annotated the negative regulation of biological processes, in addition to the same main functions as the CK vs. 2Day comparison groups. The number of upregulated proteins was significantly higher than that of downregulated proteins. This suggests that after 5 days of severe drought stress, endogenous biofeedback regulation was activated or enhanced, and the accumulation of certain substances peaked. Stimulus reversal or multiple pathways play coordinating roles (Li et al., 2022). Similarly, the functional DEPs annotation in CC and MF categories was the same as that in the CK vs. 2Day comparison group, and the number of upregulated DEPs increased greatly, including cytochrome b559α subunit, NADh-quinone REDOX reductase subunit I, photosystem II reaction centre protein H, and others. These proteins are located in chloroplasts. Additionally, they affect photosynthesis. Simultaneously, the antioxidant activity function items showed a significant expression of DEPs. The 2Day vs. 5Day comparison group was used as an example to analyse the GO entry nodes enriched by DEPs and their hierarchical relationships (**Supplementary Figure S6**). The DEPs were significantly enriched in photosynthesis (GO:0015979). The functions of these proteins included light reactions (GO:0019684) and light harvesting (GO:0009765). In addition, cellular metabolic compound salvage (GO:0043094), which can regulate the synthesis of many metabolic derivatives and provide consumption substrates, showed significant enrichment of DEPs, photorespiration assistance (GO:0009853), and a series of reactions. Simultaneously, the biological processes of anabolic products were also underway. For example, organic acid metabolic processes (GO:0006082) included carboxylic acid metabolic processes (GO:0019752) and alpha-amino acid metabolic processes (GO:1901605). Enrichment of the above functional DEPs indicated that photosynthesis and synthesis of stress-resistant substances were the main measures of plant resistance to drought under 5Day drought stress.

**Figure 11:**
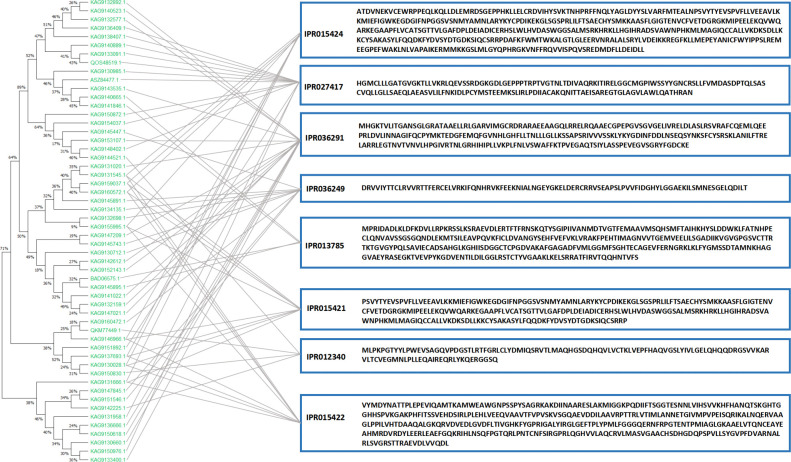
Phylogenetic tree of domain-similar DEPs.

The domains of the DEPs were annotated to further analyse their biological function and structural evolution (**Figure 9B**). The CK vs. 2Day comparison group and the 2Day vs. 5Day comparison group had similar clustering of the DEPs domain, and the main domain was a P-loop-containing nucleoside triphosphate hydrolase (IPR027417), NAD(P)-binding domain superfamily (IPR036291), thioredoxin-like superfamily (IPR036249), nucleic acid binding, and OB-fold (IPR012340). Subcellular localisation of DEPs was performed (**Figure 9C**), which were mainly distributed in the chloroplasts, cytoplasm, nucleus, and plasma membrane. The first 10 domains of the CK vs. 2Day group comparison and 2Day vs. 5Day group comparison were analysed, and there were eight common domain entries. DEPs containing these eight domains were analysed using phylogenetic analysis and DEPs with similar genetic information were identified (**Figure 11**). For example, KAG9140665.1, KAG9141846.1, KAG9159037.1, and KAG9160572.1 not only have similar domains but also similar genetic information. Although there is no exact annotation of these proteins, more molecules of the same type can be extracted according to their functional domains, gene modification, or gene synthesis using biotechnology, which will be of great value in future drought resistance and genetic research on *A. guttata* and other plants belonging to the same family. For example, proteins KAG9133091.1 and KAG9140889.1, with a similar genetic background to known proteins QOS48519.1 (note that ATP synthetase CF1β subunit), are likely to have the same function. The ATP synthetase CF1β subunit is involved in oxidative phosphorylation and photophosphorylation and catalyses the synthesis of ATP from ADP and inorganic phosphorus (Shi et al., 2001). Studying the genetic evolution of DEPs can help further analyse the relationship between DEPs and understand the evolution of key regulatory proteins under drought stress according to classification, which is conducive to further exploration at the gene level.

The biosynthetic pathway of shikonin and its derivatives, which are the main active components of *A. guttata*, is composed of three pathways (Liao et al., 2015; Suttiyut et al., 2022). The 2-C-methyl-D-erythritol-4-phosphate pathway (MEP) with pyruvate-3-phosphoglyceraldehyde as the starting substrate for the synthesis of the key substance geraniolate pyrophosphate, dimethylallyl pyrophosphate, is produced from isopentenyl pyrophosphate synthesised via the mevalonate pathway (MVA) under the action of geraniolate pyrophosphate synthase. Para-hydroxybenzoic acid and geranyl pyrophosphate from the phenylpropanoid (PP) pathway are catalysed by the key enzyme para-hydroxybenzoine-geranyl transferase to form an important precursor, geranyl para-hydroxybenzoic acid, which is then epoxidised, hydroxylated, and reacted enzymatically to produce shillin compounds. They are transported to the cell wall via exocytosecretion (Fu et al., 2020). Among these, cinnamic acid 4-hydroxylase is a key enzyme in the PP pathway, dimethyl allyl pyrophosphate isomerase is a node enzyme of the MVA pathway that regulates the synthesis of geranyl pyrophosphate, and hydroquinone geranyl-3”-hydroxylase is an important precursor catalytic enzyme for the synthesis of shikonin and directly affects the accumulation of shikonin. Ubiquinone and other terpenoid-quinone biosynthetic pathways are the main pathways involved in shikonin biosynthesis. The results showed that drought stress affected the synthesis and metabolism of shikonin and other related substances (**Supplementary Figures S7A, B**). In CK vs. 2Day, compounds such as shikonin, deoxyshikonin, isovalerylshikonin, propionylshikonin, dimethylacrylshikonin, angelylshikonin, and p-hydroxybenzoic acid were significantly upregulated, with p-hydroxybenzoic acid being the key substance for the synthesis of shikonin. Simultaneously, resistant substances, such as 3,7-di-O-methylquercetin, rosiendrobium, citric acid, xylitol, L-n-leucine, L-piperidinic acid, and jasmonyl-L-isoleucine, began to accumulate. The accumulation of these substances is involved in the regulation of flavone and flavonol biosynthesis (ko00944) and tyrosine metabolism (ko00350). Compared with 2Day of drought stress, 5Day of drought stress showed changes in plant metabolism. Shikonin was still accumulating, and the contents of shikonin, alkannin, and arnebin-4 were significantly higher than those under 2Day drought stress. However, it is worth noting that the accumulation in this period was much less than that in 2Day relative to CK, and the content of deoxyshikonin was lower than that in 2Day. The p-hydroxybenzoic acid content was only slightly higher than that of the 2Day drought stress treatment. These results show that deoxyshikonin was decomposed but not synthesised after 5Day drought stress. P-hydroxybenzoic acid is involved in the regulation of other drought stress responses, and its decomposition is accelerated, resulting in a slowdown in shikonin synthesis. After rehydration, the major effective components, such as shikonin and alkannin, did not change significantly, indicating that severe drought stress affected the synthesis and accumulation of major components and that the normal functional state may not be restored after rehydration (Xiong et al., 2022).

**Figure 12:**
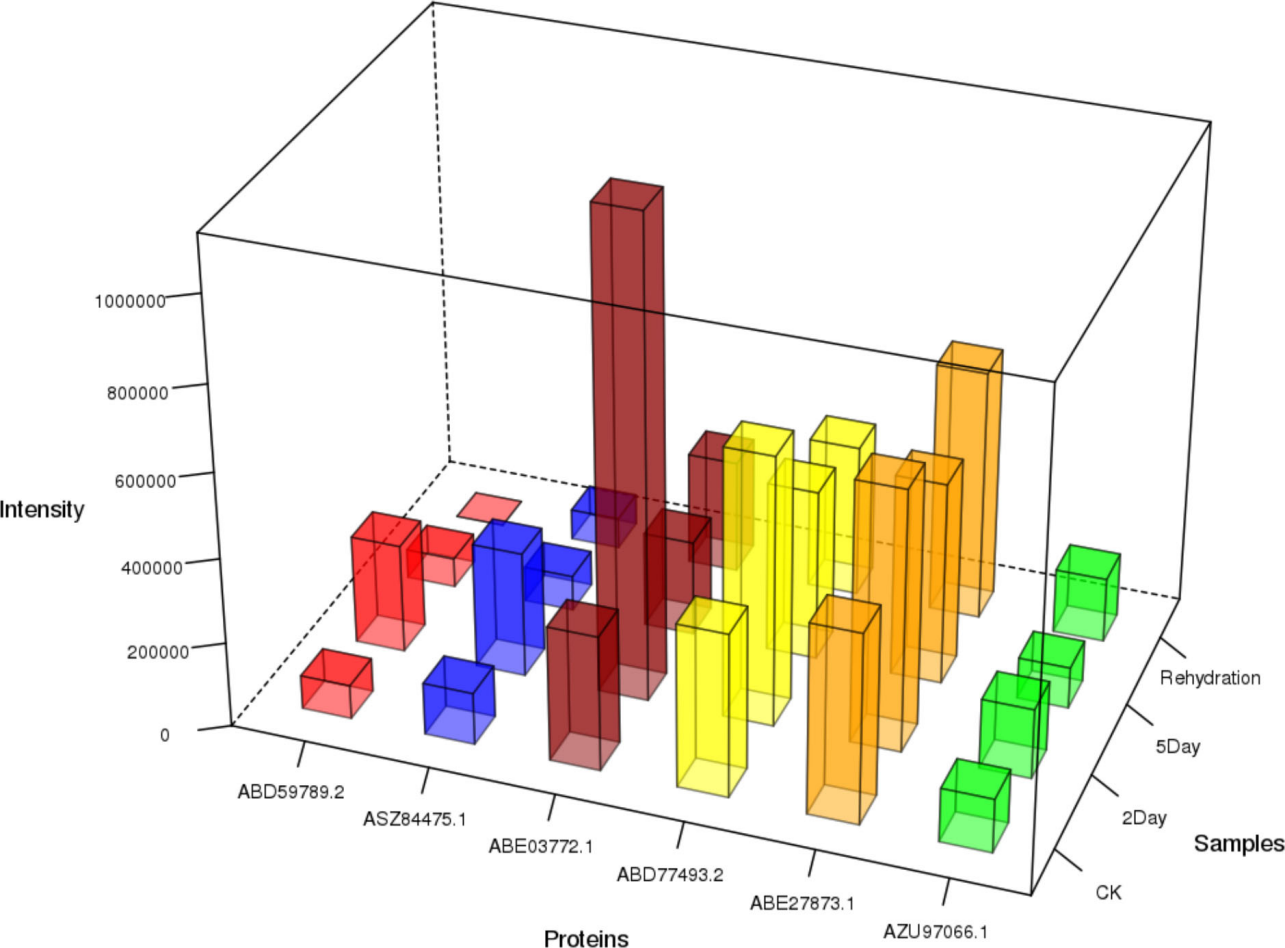
Content of shikonin-related enzymes in each group.

By analysing the correlation of expression trends between proteins and metabolites, regulatory proteins of biological significance related to key substances can be identified. Shikonin-related proteins include 4-coumaric acid:CoA ligase, 4-coumaric acid:CoA ligase 3, phenylalanine ammoniase, cinnamate-4-hydroxylase, isopentenyl pyrophosphate, dimethylallyl pyrophosphate isomerase, geranylhydroquinone 3’’-hydroxylase, and others. These proteins are involved in regulating shikoninoid synthesis and accumulation. In the CK vs. 2Day comparison group, the expressions of the key proteins in the synthesis pathway, 4-coumaric acid:CoA ligase, 4-coumaric acid:CoA ligase 3, phenylalanine ammoniase, cinnamate-4-hydroxylase, dimethylallyl pyrophosphate isomerase, and geranylhydroquinone 3’’-hydroxylase were significantly upregulated (**Figure 12**), consistent with the expressions of shikonin, deoxyshikonin and 4-hydroxybenzoic acid, indicating that the 2Day drought stress promoted the synthesis of key enzymes and led to the accumulation of bioactive substances such as shikonin. In the 2Day vs. 5Day group comparison, there was still significant accumulation of shikonin, but the extent of accumulation was much smaller than that in the CK vs. 2Day group comparison. The expression of deoxyshikonin decreased, and the expression of 4-hydroxybenzoic acid was slightly higher in 5Day drought than in 2Day drought. Simultaneously, the expression of shikonin synthases was downregulated. It is speculated that shikonin biosynthesis was no longer the main internal activity during 5Day drought but tended to resist drought stress (**Supplementary Figures S7C, D**). Simultaneously, the results of quantitative real-time PCR validation (qRT-PCR) and parallel reaction monitoring protein verification (PRM) quantitative detection also verified that the trend of related mRNA and protein expression was consistent with the results of 4D-lablefree data, which confirmed our view (**Supplementary Figures S8**, **S9**).

### 3.10 Drought stress promotes the synthesis and accumulation of phenolic acids

Phenolic acids also show different accumulation patterns under drought conditions. After 2 days of drought stress, the levels of 4-hydroxybenzoic acid, salidroside, 3-O-p-coumaryl shikimic acid, 4-cafeoyl shikimic acid, protocatechualdehyde, and trans-5-O-p-coumaryl shikimic acid increased significantly. Danshensu, 2-O-caffeoyl malic acid, coniferol, and other substances accumulated after 5 days of drought stress. The synthesis of protocatechualdehyde and danshensu as active components under drought stress conditions warrants further study. The proteins associated with protocatechualdehyde, including KAG9158682.1 and KAG9148015.1, showed the same upregulated expression trend and were also involved in the biosynthesis of shikonin. ACC oxidase 1 and LEDI-5c proteins were negatively correlated with Danshensu content. Protocatechualdehyde and Danshensu are the important active components of *Salvia miltiorrhiza*. Moderate drought stress can stimulate the activity of phenolic acid component synthetase in *S. Miltiorrhiza*, initiate the phenolic acid metabolic system, and promote the accumulation of protocatechualdehyde and danshensu (Zhou et al., 2022). The same components in different species induced the same enzymes and genes and may have the same expression trend under the same external stress.

**Figure 13:**
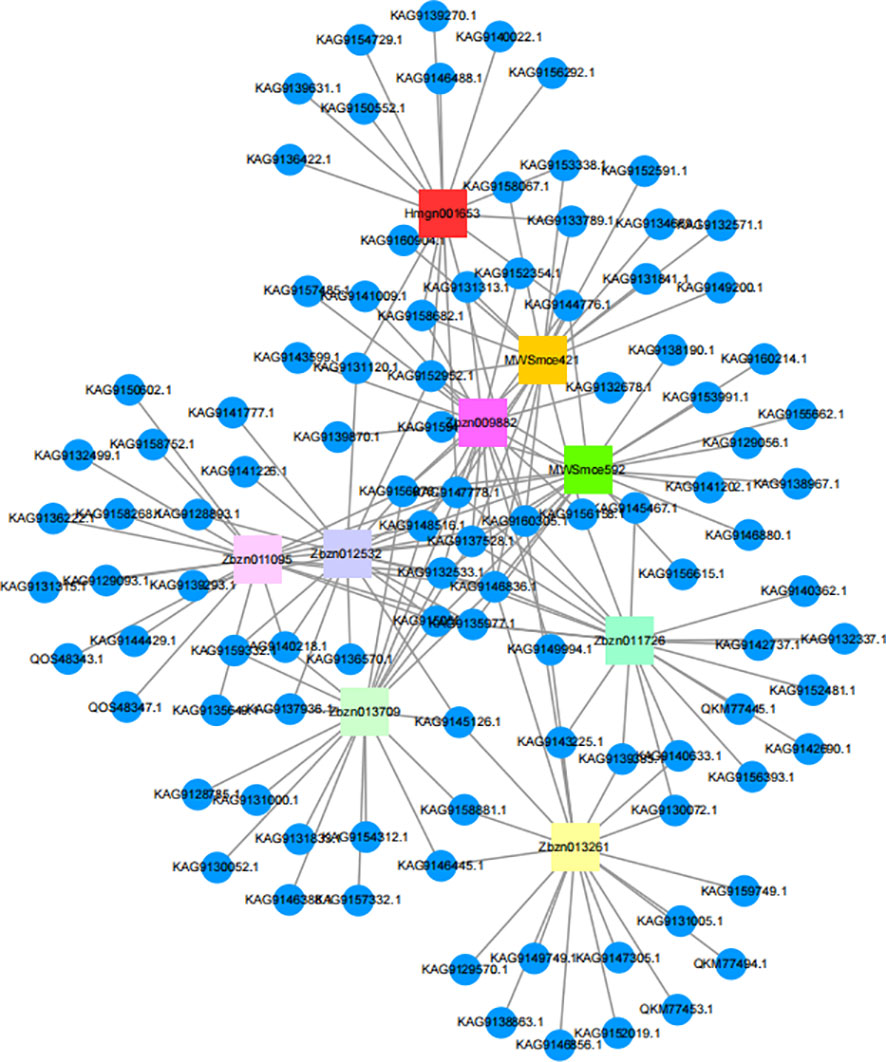
Correlation between shikonins, phenolic acids, and proteins.

The network relationships of shikonin and phenolic acids with related proteins are shown in **Figure 13**.

Analysis of the differential expression metabolites (DEMs) during 5Day drought stress revealed that the contents of alkaloids, flavonoids, organic acids, amino acids, and their derivatives increased significantly, suggesting that the plants activated and enhanced their resistance mechanisms and regulated their drought-resistant metabolic pathways to produce protective substances while maintaining normal life activities in response to severe drought stress. Therefore, the synthesis of shikonin is limited (Khandaker et al., 2022).

In response to this prediction, it was found that the protein KAG9142734.1 is a key enzyme in the synthesis of plastoquinol-9 and phenols such as (alpha-tocopherol) alpha-tocotrienol, was significantly upregulated in another branch of the synthetic pathway. Tocopherol content is positively correlated with the response of plants to drought stress. Under stress conditions, the expression of genes inducing tocopherol synthesis pathway can enhance the accumulation of tocopherol, and the increase in α-tocopherol content can reduce the level of lipid peroxidation and enhance the tolerance of plants to drought stress (Guan et al., 2016). In addition, 4-hydroxybenzoic acid is the key point of pathway branching, and its accumulation of 4-hydroxybenzoic acid regulates the negative feedback of the pathway ko00400 (phenylalanine, tyrosine, and tryptophan biosynthesis). The results showed that the expression of l-phenylalanine in the ko00400 pathway was significantly upregulated, and the enzyme KAG9155995.1 that catalysed its synthesis was also significantly increased. L-phenylalanine is a key substance in tropane, piperidine, pyridine alkaloid biosynthesis (ko00960), isoquinoline alkaloid biosynthesis (ko00950), indole alkaloid biosynthesis (ko00901), anthocyanin biosynthesis (ko00942), and other pathways. In the phenylpropane biosynthetic pathway, the content of coniferol, which can be polymerised into the natural polymer guaiac lignin, increases significantly. Lignin filling the cellulose network structure of the cell wall of land plants not only provides cell wall rigidity but also enhances the ability of plant cells and tissues to withstand other stresses. The aromatic properties determine the hydrophobicity of the cell wall, which is conducive to reducing water loss and maintaining normal expansion pressure under drought stress (Yan et al., 2021). In the anthocyanin biosynthesis pathway, the content of cyanidin 3-rutinoside chloride is significantly increased, and anthocyanins accumulate in plants during drought, low temperatures, and other stresses because anthocyanins can help plants adapt to stresses by reducing cell osmotic potential and freezing point (Muscarà et al., 2019). Therefore, anti-stress substances are highly active during the late period of drought stress, thereby maintaining metabolic processes.

### 3.11 Exploration of metabolic processes based on modular analysis

**Figure 14:**
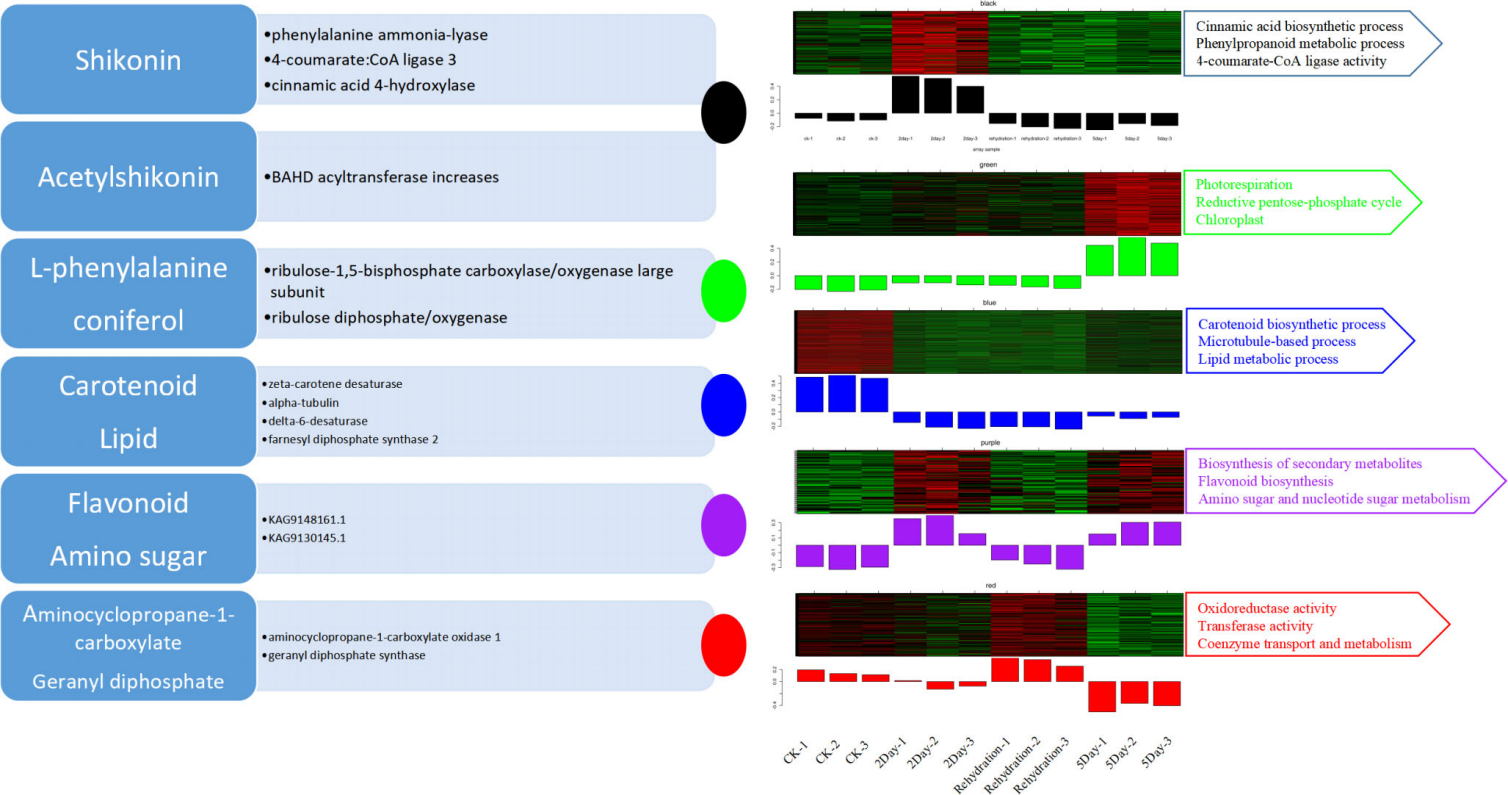
Main functional modules in the samples. The note on the left indicates the important proteins and related metabolites in each module and is distinguished by circles of different colours.

To further explore the associations between proteins and metabolites under drought stress, as well as potential regulatory factors, we performed weighted gene co-expression network analysis (WPCNA), which measures changes in protein expression in different treatment groups (**Figure 14**). Proteins with similar expression levels may be functionally related and clustered into one module. In this study, all proteins that met the criteria (proteins with low expression and no difference in expression between groups were screened) were defined into a total of 15 modules, which were represented by 15 colours. For example, proteins in the black module were significantly expressed in the 2Day drought stress group, mainly phenylalanine ammonia lyase, 4-coumarate:CoA ligase 3, cinnamic acid 4-hydroxylase, and other proteins associated with shikonin synthesis. It is worth noting that the content of BAHD acyltransferase increased, which is an essential enzyme for the synthesis of acylation modifications of plant secondary metabolites, especially in the conversion of the shikonin derivative acetylshikonin. Specific BAHD acyltransferases recognise acetyl-CoA, isobutylkylase A, and isoglutarate CoA as acyl donors to produce the corresponding shikolin derivatives (Oshikiri et al., 2020). The kWithin (connectivity of proteins within modules) values of these related enzymes were all in the top rank, and they were the core proteins in the black module. Their expression levels increased, which was consistent with the increasing trend of shikonin content in the metabolic data. The ribulose-1,5-bisphosphate carboxylase/oxygenase large subunit is the core protein in the green module which significantly accumulated in the 5Day drought stress group. Theoretically, under severe drought stress for 5 days, the activity of the ribulose-1,5-bisphosphate carboxylase/oxygenase large subunit should be weakened because of the reduced electron transfer efficiency of photosynthesis (Zhang et al., 2023), but the results were the opposite. It has been speculated that the interior of plants responds positively to severe drought stress. In addition, the content of carboxylase of ribulose diphosphate/oxygenase activator also increased, and their strongly related metabolites, L-phenylalanine and coniferol, are important stress-resistant substances (Chen et al., 2020; Qin et al., 2022), their contents were the highest under 5Day drought stress and jointly regulate the influence of drought.

In the blue module, proteins, including zeta-carotene desaturase, alpha-tubulin, delta-6-desaturase, and farnesyl diphosphate synthase 2, were highly expressed in the CK group. These proteins are involved in carotenoid biosynthesis, chloroplast biocomposition, microtubule-based processes, and lipid metabolism. Under normal water supply, plants have these normal functions; however, under drought stress, the expression of related proteins is inhibited, especially in the 5Day drought stress group.

The proteins in the purple module showed high expression in the drought stress groups (2Day and 5Day) and strong inhibition of expression in the CK and Rehydration groups. They are involved in the biosynthesis of secondary metabolites, flavonoids, amino sugars, nucleotide sugars and other metabolic pathways. The results showed that the normal environment was suitable for plant growth and development. In contrast, under environmental stress, plants accumulate secondary substances, including flavonoids and alkaloids (Naliwajski et al., 2022).

The expression of some proteins decreased with the deepening of drought and was concentrated in the red module. Two of these proteins, namely aminocyclopropane-1-carboxylate oxidase 1 (ACO1) and geranyl diphosphate synthase (GPPS), deserve attention. Both genes were downregulated under drought stress and the degree of downregulation was proportional to the degree of drought stress. ACO1 regulates ethylene synthesis in plants under abiotic stresses, such as salt stress. Ethylene signalling plays an important role in plant growth and development. ACO1 catalysed aminocyclopropane-1-carboxylate (ACC) conversion to ethylene via negative regulation (Chen et al., 2014). The results showed that drought stress inhibited the expression of ACO1, promoted ethylene production, and improved drought tolerance. GPPS are key enzymes in the synthesis of shikonin derivatives and are responsible for providing substrates for alkenyl transferases (Ueoka et al., 2020). Unlike other regulatory proteins involved in shikonin synthesis, GPPS catalyses geranyl diphosphate (GPP) synthesis through negative regulation under drought stress. Thus, the regulation of enzymes involved in shikonin synthesis under drought stress was analysed (**Figure 10**).

These results indicate that additional metabolic regulation strategies under drought stress can be explored using modular analysis.

## 4 Discussion

### 4.1 *A. guttata* quality is closely related to climate

BIO18 was the largest contributor among climate factors to the national distribution of *A. guttata*, and precipitation from May to September was closely related to the suitability distribution of *A. guttata*. Flowering and fruiting periods of *A. guttata* occur from June to September, and precipitation during the warmest season has a significant effect on its growth and metabolism. After resource investigation, we found that the abundance of *A. guttata* in Xinjiang was low, and the corresponding climatic phenomena were characterised by low precipitation. It has been speculated that the growth and development of *A. guttata* are limited by a lack of rainfall, drought, and poor habitat. In Inner Mongolia, precipitation during the warmest season in Bayanbaolig Town, Wulat Back Banner, was in the middle of all sample points, and *A. guttata* was the most abundant. There was a correlation of precipitation and sample distribution, and the area with moderate rainfall was more suitable for the growth of *A. guttata*. Using multi-omics, phylogenetic analysis, and laboratory verification, a major scientific matter was solved: what is the relationship between soil drought and quality formation owing to rainfall differences. For example, ginsenosides are the active ingredients in *Panax ginseng*, and their content affects the quality of the derived medicinal materials. Previous studies have shown that the soil moisture content affects ginsenoside synthesis and accumulation. Moderate drought can increase ginsenoside content, but long-term drought can inhibit the growth of roots (Lei et al., 2023). *Codonopsis pilosula* is mainly distributed in arid and semi-arid areas in northwest China. Studies on drought stress and synthesis of medicinal components showed that moderate drought was conducive to the accumulation of polysaccharides and codonopsis acetylene glycosides in the above ground and roots of *C. pilosula*. Severe drought promotes the biosynthesis of lipid III in atractyla, indicating that drought stress has a significant effect on the accumulation of metabolites of *C. pilosula* (Sun et al., 2022). In addition, it was found that under mild drought stress, a large number of flavonoids accumulated significantly in *Sophora alopecuroides*, but under moderate drought stress, the flavonoid abundance decreased, the expressions of related flavonoid biosynthesis genes IF7GT and IF7MAT were downregulated, and the lipid abundance increased (Huang et al., 2023). These studies have shown that drought significantly affects the accumulation of active ingredients in medicinal materials. In this study, the shikonin content in the samples from Daban City of Urumqi and Bayanbaolige Town of Urat Houqi of Bayannur City was the highest; however, the degree of drought in the two regions was different. The precipitation in the warmest season in Daban City of Urumqi was low, the precipitation in Bayanbaolig town in Urat Houqi of Bayannur City had a median value, shikonin content in the samples of Baijigou Street in Dawukou District of Ishuishan City was the lowest, and the precipitation in the warmest season was also the highest. It can be seen from the analysis of this rule that under drought stress, the accumulation of shikonin substances in *A. guttata* increased significantly, and the content of active substances under severe drought stress was high, but there was no significant difference when compared to that under moderate drought stress. In contrast, because of its low water content, plant growth was inhibited, which was not conducive to its survival and affected its yield. When plants suffer from a severe water deficit, metabolite synthesis gradually transforms into primary metabolism to maintain normal physiological activities (Tiedge et al., 2022). Therefore, moderate drought stress is conducive to increasing the quality of *A. guttata*.

### 4.2 Metabolic diversity reveals the environmental adaptability of *A. guttata*

The metabolic richness improved the adaptability of *A. guttata* to a drought environment. With interactions with climate, the regulatory relationship between gene proteins and material metabolism has changed significantly, resulting in more tolerant life strategies. Shikonin is the main active ingredient in *A. guttata*. Moderate drought stress is beneficial to the regulation of proteins 4-coumarate-coenzyme A ligase, cinnamic acid 4-hydroxylase, phenylalanine ammonia lyase, 4-coumarate-coenzyme A ligase 3, cinnamic acid-4-hydroxylase, dimethyl allyl pyrophosphate isomerase, hydroquinone-3”-hydroxylase, and others, and increases the accumulation of 4-hydroxybenzoic acid, deoxyshikonin, and shikonin. Severe drought stress inhibits the normal growth and development of *A. guttata*, which is not conducive to the continuous synthesis of medicinal components and affects their medicinal functions. Phenolic acids, including protocatechualdehyde and danshensu, and its related proteins KAG9158682.1, KAG9148015.1, ACC oxidase 1, and LEDI-5c, regulate substance accumulation through different regulatory modes. KAG9158682.1 and KAG9148015.1 also regulate shikonin biosynthesis. The drought resistance of plants is reflected not only in the degree of drought resistance but also in their ability to recover normal growth after rehydration (Han et al., 2022), indicating the *A. guttata* has some drought resistance. During drought, the antioxidant enzyme system plays a role in scavenging ROS and improving *A. guttata* to cope with drought stress. When seedlings are subjected to drought stress, many free radicals and hydrogen peroxide are produced in the cells, and lipid peroxidation of the cell membranes intensifies, damaging the cell membrane system (Nadeem et al., 2021). Under these circumstances, the activities of enzymes, including CAT, POD, and SOD, increase, helping remove internal toxic substances. ROS within plants are in a state of dynamic equilibrium. Zhuang et al. (2021) showed that, under certain abiotic stress conditions, certain genes can regulate the balance of reactive oxygen species in plants by affecting the rate of H_2_O_2_ production in chloroplasts. Therefore, it is highly likely that fluctuations in the levels of reactive oxygen species within plants can lead to corresponding fluctuations in the levels of endogenous antioxidant enzymes. For instance, Jian et al. (2021) found that in tomatoes subjected to simulated drought stress, the activities of SOD and POD peaked at 24 h and then declined by 72 h. SOD can convert excess oxygen free radicals into H_2_O_2_. POD and CAT can convert H_2_O_2_ into O_2_, and the three enzymes protect each other and play a synergistic role (Khaleghi et al., 2019). ABA regulates plant responses to drought stress by inducing enzymes and genes related to cellular dehydration tolerance (Vonapartis et al., 2022). ABA is a stress hormone that helps plants adapt to external environmental stress and improves their drought tolerance (Perin et al., 2019). Chlorophyll is the primary component of plant photosynthesis. Drought stress restricts the metabolism and physiological processes of plants, inhibits chlorophyll synthesis, accelerates the decomposition rate, and reduces chlorophyll content (Demirkol, 2021). However, the results of these experiments showed that the chlorophyll content increased significantly under drought stress and reached its peak after the 2Day drought stress. An increase in chlorophyll content can improve a plant’s adaptability to drought stress. Under moderate drought conditions, the synthesis rate of chlorophyll was higher than the decomposition rate, the photosynthetic capacity was strengthened, and the synthesised assimilated organic matter was preferentially assigned to the root position. It increased the mass of root dry matter, thereby increasing the amounts of metabolites. However, 5Day drought stress was severe in the seedlings. At this time, the chloroplast membrane structure is destroyed, which directly leads to a reduction in chlorophyll synthesis and photosynthesis and inhibits the overall growth and development of *A. guttata* (Cao et al., 2021; Zhu et al., 2021). Pro is a free osmotic regulatory substance. Cells reduce their intracellular osmotic potential by accumulating osmotic regulatory substances to ensure normal intracellular turgor pressure and water absorption capacity to adapt to drought (Huang et al., 2022). The effective regulation of Pro content indicated that *A. guttata* has a strong ability to resist dehydration and drought stress. Drought stress increases membrane lipid peroxidation and the accumulation of MDA. The degree of response of MDA plants to stress is also defined as the degree of damage caused by the stress. When plants are subjected to drought stress, the content of MDA generally shows a steady increase. In short, plants with a small increase in MDA content are more tolerant to drought, whereas those with a large increase in MDA content are less tolerant to drought (Wang Y. et al., 2022). The changes in ABA, MDA, Pro and chlorophyll contents indicate that it can self-regulate a certain degree of drought. Ribulose diphosphate carboxylase/oxygenase-activating enzymes are involved in the synthesis of flavonoids and alkaloids, which play important regulatory roles under severe drought stress.

4-Coumarate-CoA ligase is a terminal enzyme in the downstream branch of the phenylpropane metabolic pathway (Tian et al., 2017). Phenylalanine ammonia lyase is the starting enzyme of the PP pathway, which may be involved in the synthesis of shikonin (Wang X. et al., 2022), cinnamic acid-4-hydroxylase, dimethylallyl pyrophosphate isomerase, and hydroquinone-3”-hydroxylase are key regulatory enzymes involved in shikonin synthesis. The phenylpropane metabolic pathway, which provides p-hydroxybenzoic acid necessary for shikonin synthesis, is a key pathway in shikonin biosynthesis. At the gene level, 4-coumarate:CoA ligase mRNA, 4-coumarate:CoA ligase 3 mRNA, ribulose bisphosphate carboxylase/oxygenase activase (RCA) mRNA, N-carbamoylputrescine amidase (LOC115678090) mRNA, and other protein-coding genes showed significant positive regulatory correlations with key proteins. The gene sequences of 4-coumaric-CoA ligase, 4-hydroxylase of cinnamic acid, and phenylalanine ammonlyase have been identified and cloned from *Lithospermum erythrorhizon* (Yazaki et al., 1995; Yazaki et al., 1997). In terms of stress resistance, flavonoids, alkaloids, organic acids, sucrose, amino acids and their derivatives accumulated significantly, and related proteins, including ribulose diphosphate carboxylase/oxygenase activase, were actively expressed during drought regulation. The accumulation of flavonoids and other substances can improve the antioxidant capacity of plants to respond to drought stress and, at the same time, the increase of amino acids, sucrose and other contents help plants resist the damage caused by drought stress (Li et al., 2020). There were different evolutionary relationships among the differentially functional genes in samples from the three locations. Cluster-164174.141068 and Defensin SD2 (Cluster-164174.160237), transaldolase-like (Cluster-164174.177972) and cytochrome P450 monooxygenase psoD-like (Cluster-225483.5), B2 protein-like (Cluster-164174.158171) and albumin-1 (Cluster-164174.106959), ERF transcription factor (Cluster-164174.124558) and transcription factor bHLH148-like (Cluster-164174.117707), and other differential genes have close relatives or homologous genes. ACC oxidase 1,4-coumarate:CoA ligase (Cluster-164174.168700) and cytochrome P450 monooxygenase psoD-like were key functional genes, which regulate the synthesis of shikonins and phenolic acids. The ERF and bHLH families are closely related to the development of transcription factors and play important roles in drought resistance. Owing to the external climate, their expression differs among samples from different production areas. Phylogeny revealed the genetic evolution of the differential functional genes in the samples from the three locations and found functional genes that were homologous or closely related, which could be used as target genes to trace more functional genes and expand the functional gene dataset. Simultaneously, their expression in different samples was analysed to explore the correlation between differential gene expression and climate in different samples. In the future, gene sequences may be modified using genetic technology to optimise provenance and improve drought tolerance and effective component accumulation in *A. guttata*.

### 4.3 Drought stress and future development of suitable areas for *A. guttata*

In the next 40 years, suitable areas for *A. guttata* will be distributed in the eastern part of Inner Mongolia, and the degree of drought in the eastern part of Inner Mongolia will be moderate. The results of this study indicate that the natural distribution of *A. guttata* was closely related to the degree of drought. Among the collections from three cultivation areas, the distribution of *A. guttata* in Xinjiang was the lowest, and the densest area was approximately 200 plants/km^2^. During the warmest season in Xinjiang, precipitation is low, the degree of drought is strong, and environmental conditions are harsh. Although the total content of the effective components of the samples was slightly higher in some areas, there was no significant difference. In addition, living conditions limited growth and development, resulting in low yields and uneven quality. In Inner Mongolia, the distribution is the densest (approximately 1000 plants/km^2^), and the total content of effective components and the yield are high. Eastern Inner Mongolia is the main distribution area of *A. guttata*. Suitable drought conditions not only affect growth but also promote the accumulation of effective components. Under future conditions of a stable climate, Eastern Inner Mongolia will become the main growth area, which provides a reference for future artificial cultivation.

## Data availability statement

The Proteomic data of *Arnebia guttata *presented in the study are deposited in the iProX repository, accession number PXD052741. Rhizosphere soil microbial data presented in the study are deposited in the NCBI repository, accession number PRJNA1118909. Transcriptome data of wild samples of *Arnebia guttata* presented in the study are deposited in the NCBI repository, accession number PRJNA1118926.

## Author contributions

QL: Visualization, Writing – original draft, Data curation. HL: Writing – review & editing. MZ: Writing – original draft, Data curation. GL: Writing – original draft, Data curation. ZZ: Writing – original draft, Data curation. XC: Writing – original draft, Investigation. XW: Writing – original draft, Data curation. CZ: Writing – original draft, Resources, Methodology, Conceptualization. ML: Writing – review & editing, Writing – original draft, Supervision, Resources, Methodology, Conceptualization.

## Supplementary material

Supplementary Figure 1 Distribution of BIO18 at 18 sample points. From red to blue, the value of BIO18 increased gradually.

Supplementary Figure 2 Distribution of genes in different samples **(A)**. Correlation between samples **(B)**. PCA of samples from different provinces

Supplementary Figure 3 Sample hierarchical clustering tree.

Supplementary Figure 4 LDA distribution histogram of rhizosphere bacterial communities from different producing areas.

Supplementary Figure 5 PCA analysis **(A)**, and DEPs cluster heat map **(D)**.

Supplementary Figure 6 GO enrichment circle diagram **(A)**, and directed acyclic graph of enriched GO terms **(B).**

Supplementary Figure 7 K-Means map of differential metabolites **(A)**, metabolite difference abundance score map **(B)**, Correlation analysis of nine quadrants **(C)**, and CCA **(D)**.

Supplementary Figure 8 Validation of qRT-PCR. The left Y axis is the QPCR coordinate, and the right Y axis is the proteomic coordinate.

Supplementary Figure 9 Validation of PRM.
