# Supplementary material for: Multifaceted insights into the environmental adaptability of Arnebia guttata under drought stress
Source: Front Plant Sci. 2024 Jun 13;15:1395046. doi: 10.3389/fpls.2024.1395046 (PMC11210590; doi:10.3389/fpls.2024.1395046)
Supplement: Supplementary file 1 [file Table_1.docx]

Table S1. Sample information of *A. guttata*.

| **No.** | **Longitude(°E)** | **Latitude(°N)** | **Collection area** |
| --- | --- | --- | --- |
| A1 | 87.9436 | 43.6067 | Urumqi Daban city |
| A2 | 89.3524 | 43.1953 | Gaochang District, Turpan City |
| A3 | 93.7938 | 43.1443 | Yizhou District, Hami City |
| B1 | 106.5130 | 39.3887 | Alxa League Alxa left banner Zong Bili town |
| B2 | 105.7050 | 38.6822 | Alxa League Alxa left banner Bayanhot town |
| B3 | 106.5545 | 39.4176 | Alxa League Alxa left banner Zong Bili town |
| B4 | 106.3349 | 40.5914 | Shajin Tao Hai Sumu, Dengkou County, Bayan Nur City |
| B5 | 107.0521 | 41.0885 | Bayanbaolig Town, Urat Houqi, Bayannur City |
| B6 | 107.0650 | 41.2704 | Yin Mountain, Urat Houqi, Bayan Nur City |
| B7 | 107.0922 | 41.2368 | Yin Mountain, Urat Houqi, Bayan Nur City |
| B8 | 107.0782 | 41.2438 | Yin Mountain, Urat Houqi, Bayan Nur City |
| B9 | 107.0624 | 41.2489 | Yin Mountain, Urat Houqi, Bayan Nur City |
| C1 | 105.2273 | 37.4627 | Zhongwei City Shapotou District Yongkang Town |
| C2 | 105.2068 | 37.3254 | Xiangshan Township, Shapotou District, Zhongwei City |
| C3 | 105.2584 | 37.0037 | Xingren Town, Shapotou District, Zhongwei City |
| C4 | 105.7754 | 38.0902 | Qujing Town, Qingtongxia, Wuzhong City |
| C5 | 105.7988 | 38.0515 | Qujing Town, Qingtongxia, Wuzhong City |
| C6 | 106.2519 | 38.9432 | Baijigou Street, Dawukou District, Shizuishan City |

Annotation: A for Xinjiang, B for Inner Mongolia, and C for Ningxia. In Xinjiang, samples were found in Chaiwopu of Daban District of Urumqi,Yar Town of Gaochang District of Turpan, Keilaoba Town of Tokxun County of Turpan, Xialaoba Town of Balikun Kazakh Autonomous County of Hami, and Bizhan Town of Shanshan County of Yizhou District, Hami. In Inner Mongolia, samples were found in Zongbili Town of Alxa Left Banner of Alxa League, Bayanhot Town of Alxa Left Banner of Alxa League, Shajintao Haisumu Town of Dengkou County of Bayan Nur City, and Bayan Baolige Town of Urathoubanner of Bayan Nur City. Although Minqin County of Wuwei City, Shandan County of Zhangye City, Xintian Town of Minle County, Liuba Town of Minle County, and Heli Town of Gaotai County were also considered and visited for sampling, no plant samples could be collected. Finally, additional plant samples were found in Yongkang Town, Xiangshan Town, and Xingren Town of Shapotou District of Zhongwei City; Qujing Town of Qingtongxia City; and Baijigou Street of Dawukou District of Shizuishan City. Based on the abundance and morphological similarity of local A. guttata samples, selective collection and recording were conducted.

Table S2. Bio18 value of each sample point.

| **No.** | **BIO18（mm）** |
| --- | --- |
| A1 | 30.285 |
| A2 | 19.179 |
| A3 | 7.314 |
| B1 | 196.884 |
| B2 | 188.031 |
| B3 | 197.105 |
| B4 | 149.761 |
| B5 | 151.814 |
| B6 | 145.719 |
| B7 | 147.449 |
| B8 | 146.883 |
| B9 | 146.619 |
| C1 | 186.993 |
| C2 | 190.267 |
| C3 | 203.286 |
| C4 | 197.859 |
| C5 | 198.869 |
| C6 | 200.422 |

Table S3. Anosim analysis

| **Group** | **Sample size** | **Permutations** | **R** | **P-value** |
| --- | --- | --- | --- | --- |
| A vs B vs C | 108 | 200 | 0.4174 | 0.005 |
| A vs B | 72 | 200 | 0.5037 | 0.005 |
| A vs C | 54 | 200 | 0.7456 | 0.005 |
| B vs C | 90 | 200 | 0.2517 | 0.005 |

Table S4. MetaStat analysis

| **Level** | **Taxa** | **A-vs-B** | **B-vs-C** | **A-vs-C** |
| --- | --- | --- | --- | --- |
| Phylum | *Actinobacteriota* | 9.99E-04 | 1.30E-02 | 9.99E-04 |
|  | *Proteobacteria* | 9.99E-04 | 4.50E-02 | 9.99E-04 |
|  | *Chloroflexi* | 9.99E-04 | 9.99E-04 | 9.99E-04 |
|  | *Bacteroidota* | 9.99E-04 | 9.99E-04 | 9.99E-04 |
| Class | *Bacteroidia* | 9.99E-04 | 9.99E-04 | 9.99E-04 |
|  | *Rubrobacteria* | 9.99E-04 | 9.99E-04 | 9.99E-04 |
|  | *Chloroflexia* | 9.99E-04 | 4.50E-02 | 9.99E-04 |
| Order | *Micrococcales* | 4.00E-03 | 2.00E-03 | 8.99E-03 |
|  | *Rhodobacterales* | 9.99E-04 | 9.99E-04 | 9.99E-04 |
|  | *Rubrobacterales* | 9.99E-04 | 9.99E-04 | 9.99E-04 |
|  | *Gaiellales* | 3.60E-02 | 9.99E-04 | 9.99E-04 |
| Family | *Micrococcaceae* | 3.00E-03 | 8.99E-03 | 9.99E-04 |
|  | *Rhodobacteraceae* | 9.99E-04 | 9.99E-04 | 9.99E-04 |
|  | *Rubrobacteriaceae* | 9.99E-04 | 9.99E-04 | 9.99E-04 |
|  | *Longimicrobiaceae* | 9.99E-04 | 1.40E-02 | 2.00E-03 |
| Genus | *Rubrobacter* | 9.99E-04 | 9.99E-04 | 9.99E-04 |
|  | *Rubellimicrobium* | 9.99E-04 | 1.30E-02 | 9.99E-04 |

Table S5. Soil water content.

| **Treatment** | **Mean water content（%）** |
| --- | --- |
| CK | 35.68 |
| 2Days | 30.91 |
| 5Days | 16.44 |
| Rehydration | 32.77 |

Table S6. Primer design.

| **Gene annotation information** | **Primer sequence** |
| --- | --- |
| Internal reference gene ACT7 | Forward: 5'-TCCCTGGTATTGCTGATCGT-3' |
|  | Reverse: 5'-ACTTCCGGTGGACAATGGAT-3' |
| 4-coumarate:CoA ligase mRNA | Forward: 5'-AACTGGTGTCTCTTTGCCTCGTAAC-3' |
|  | Reverse: 5'-GTCGCCTCGGGATCATTCAGATAAC-3' |
| 4-coumarate:CoA ligase 3 mRNA | Forward: 5'-ATTGGCTATGTGGACGACGATGATG-3' |
|  | Reverse: 5'-TGTTGGTGTGGGAGATGAGGAGAG-3' |
| ribulose bisphosphate carboxylase/oxygenase activase (RCA) mRNA | Forward: 5'-ACGGGAAGGCAGCACAACAAC-3' |
|  | Reverse: 5'-CTACCATCATCACTCCTCGCTGTTG-3' |
| N-carbamoylputrescine amidase (LOC115678090), mRNA | Forward: 5'-CGCAGGACCAACGGGAGAAATC-3' |
|  | Reverse: 5'-TAGCCAGATCAAATTCCGCCACAAG-3' |
| phenylalanine ammonia-lyase mRNA | Forward: 5'-ACACTACCACACTCAGCAACAAG-3' |
|  | Reverse: 5'-GGCACGAGGTCACCAGAGG-3' |
| ribulose-1,5-bisphosphate carboxylase/oxygenase large subunit (rbcL) gene | Forward: 5'-ATCTTGGCAGCATTCCGAGTAAC-3' |
|  | Reverse: 5'-CCGTTGTCCATGTACCAGTAGAAG-3' |
| PetB (petB) gene | Forward: 5'-TACTATCGTCCGACCGTTACTGAG-3' |
|  | Reverse: 5'-GACCATCATACTTGCCGACCATC-3' |
| ATP synthase CF0 B subunit (atpF) gene | Forward: 5'-AAGTGGAAATGGAAGCCGATCAG-3' |
|  | Reverse: 5'-GCTTGTTGGAAAACCCGTTGTC-3' |
| NdhJ (ndhJ) gene | Forward: 5'-TCGTTTGTCTGCTTGGCTAGTAAAG-3' |
|  | Reverse: 5'-GCACATTGGGAGCGTAGATAGTTG-3' |
| NADH-plastoquinone oxidoreductase subunit I protein (ndhI) gene | Forward: 5'-AACAATACGAGCAGCCAGGTAC-3' |
|  | Reverse: 5'-GAACACATACTTCGCAAGCAATG-3' |
| Arnebia euchroma cinnamic acid 4-hydroxylase mRNA | Forward: 5'- TGGTCCATTGAGTGGGGCATTG-3' |
|  | Reverse: 5'-TGTACGCCAGGTCCAAGAATCG-3' |
| Arnebia euchroma isopentenyl pyrophosphate:dimethyllallyl pyrophosphate isomerase mRNA | Forward: 5'-GGTGAATCCGAACCCTGACGAG-3' |
|  | Reverse: 5'-CCACGATGAGACGGAACCAAGG-3' |
| Lithospermum erythrorhizon mRNA for LEDI-5b protein | Forward: 5'-CTGACACTGCCCAAGGATACCC-3' |
|  | Reverse: 5'-AACTCTCCCAACGTGCCACAG-3' |

Table S7. The data of the physiological and biochemical index.

| **Grouping variables** | | **Dependent variables** | | | | | | |
| --- | --- | --- | --- | --- | --- | --- | --- | --- |
| group | | CAT(U/mL) | SOD(U/L) | POD(mU/L) | ABA(ng/L) | Chlorophyll(pmol/L) | Pro(ng/L) | MDA(nmol/L) |
| 1 | | 2.011057 | 1792.5 | 10.81395 | 216.7073 | 53.0102 | 893.75 | 2.424033 |
| 1 | | 2.087077 | 1840 | 10.9034 | 203.5366 | 59.23469 | 900 | 2.564457 |
| 1 | | 2.07671 | 1945 | 12.32558 | 237.439 | 62.95918 | 945 | 2.605893 |
| 2 | | 1.997236 | 2337.5 | 14.40072 | 230.4878 | 74.84694 | 1062.5 | 2.854512 |
| 2 | | 2.280581 | 2310 | 14.48122 | 276.8293 | 75.2551 | 923.75 | 2.960405 |
| 2 | | 1.986869 | 2422.5 | 14.52594 | 260.9756 | 82.60204 | 890 | 3.190608 |
| 3 | | 2.47754 | 1832.5 | 13.2737 | 216.8293 | 60.15306 | 1085 | 2.955801 |
| 3 | | 2.252937 | 1962.5 | 12.90698 | 220.8537 | 61.12245 | 1092.5 | 2.8407 |
| 3 | | 2.211472 | 2010 | 12.2093 | 248.1707 | 67.34694 | 1150 | 3.135359 |
| 4 | | 1.897028 | 1992.5 | 10.9839 | 245.7317 | 66.78571 | 883.75 | 2.661142 |
| 4 | | 2.256393 | 1860 | 11.85152 | 214.5122 | 74.08163 | 872.5 | 2.909761 |
| 4 | | 2.39461 | 2002.5 | 11.8873 | 257.6829 | 72.95918 | 710.5 | 3.059392 |
| shapiro test | 1 | 0.2408 | 0.5906 | 0.1008 | 0.7554 | 0.7248 | 0.2139 | 0.4185 |
|  | 2 | 0.05941 | 0.452 | 0.6877 | 0.6556 | 0.08934 | 0.3546 | 0.5982 |
|  | 3 | 0.2776 | 0.4993 | 0.6608 | 0.2259 | 0.2377 | 0.2018 | 0.7601 |
|  | 4 | 0.5203 | 0.1201 | 0.0668 | 0.5183 | 0.2738 | 0.1109 | 0.7278 |
| levene test | / | 0.6713115 | 0.9759829 | 0.7270966 | 0.96601 | 0.9862075 | 0.7614057 | 0.8516114 |
| bartlett test | / | 0.2639 | 0.9552 | 0.0949 | 0.9621 | 0.986 | 0.3358 | 0.829 |
