# Supplementary figures and images for: Multifaceted insights into the environmental adaptability of Arnebia guttata under drought stress
Source: Front Plant Sci. 2024 Jun 13;15:1395046. doi: 10.3389/fpls.2024.1395046 (PMC11210590; doi:10.3389/fpls.2024.1395046)

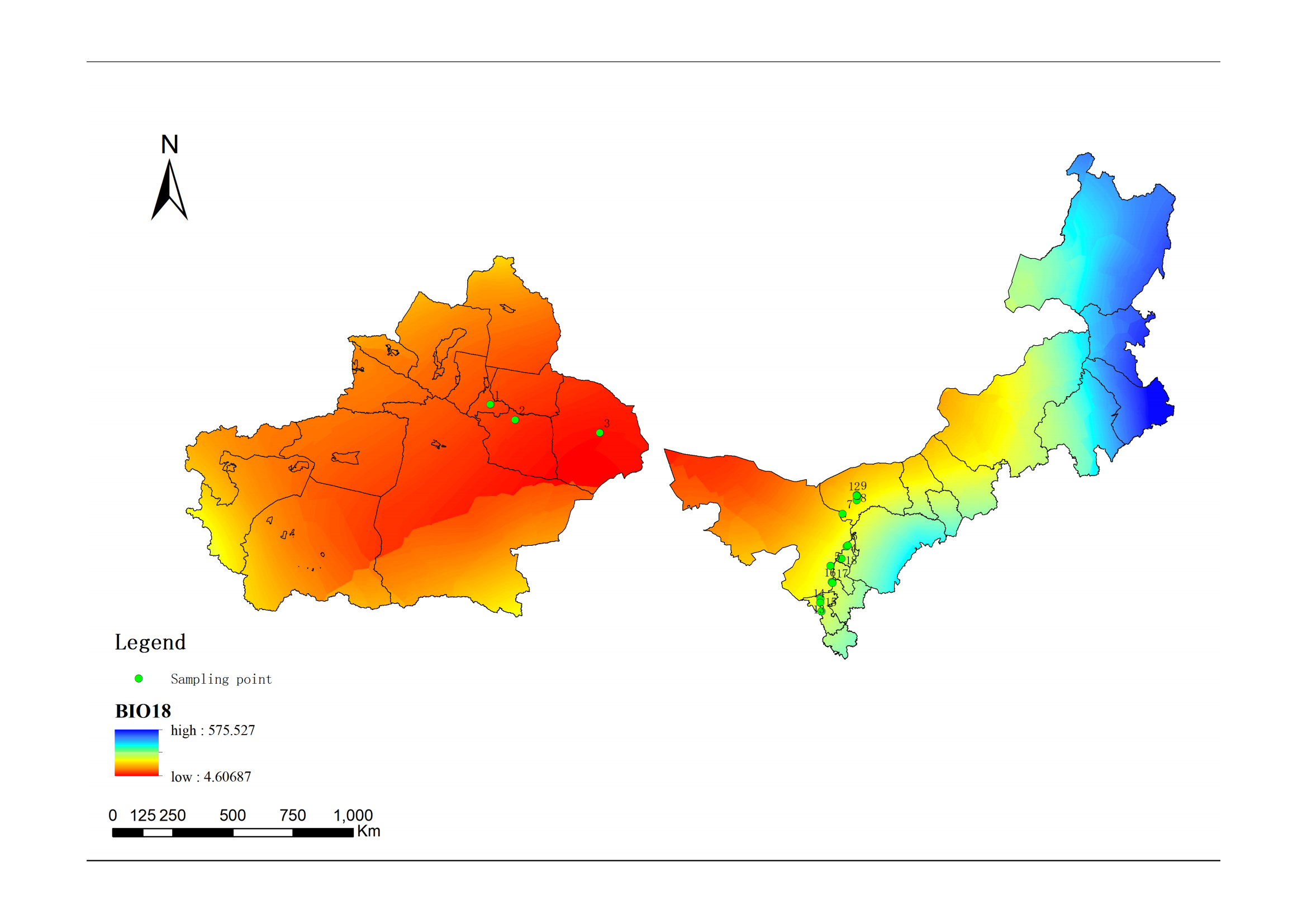

Supplement: Supplementary Figure 1 — Distribution of BIO18 at 18 sample points. From red to blue, the value of BIO18 increased gradually. [file Image_1.tif]

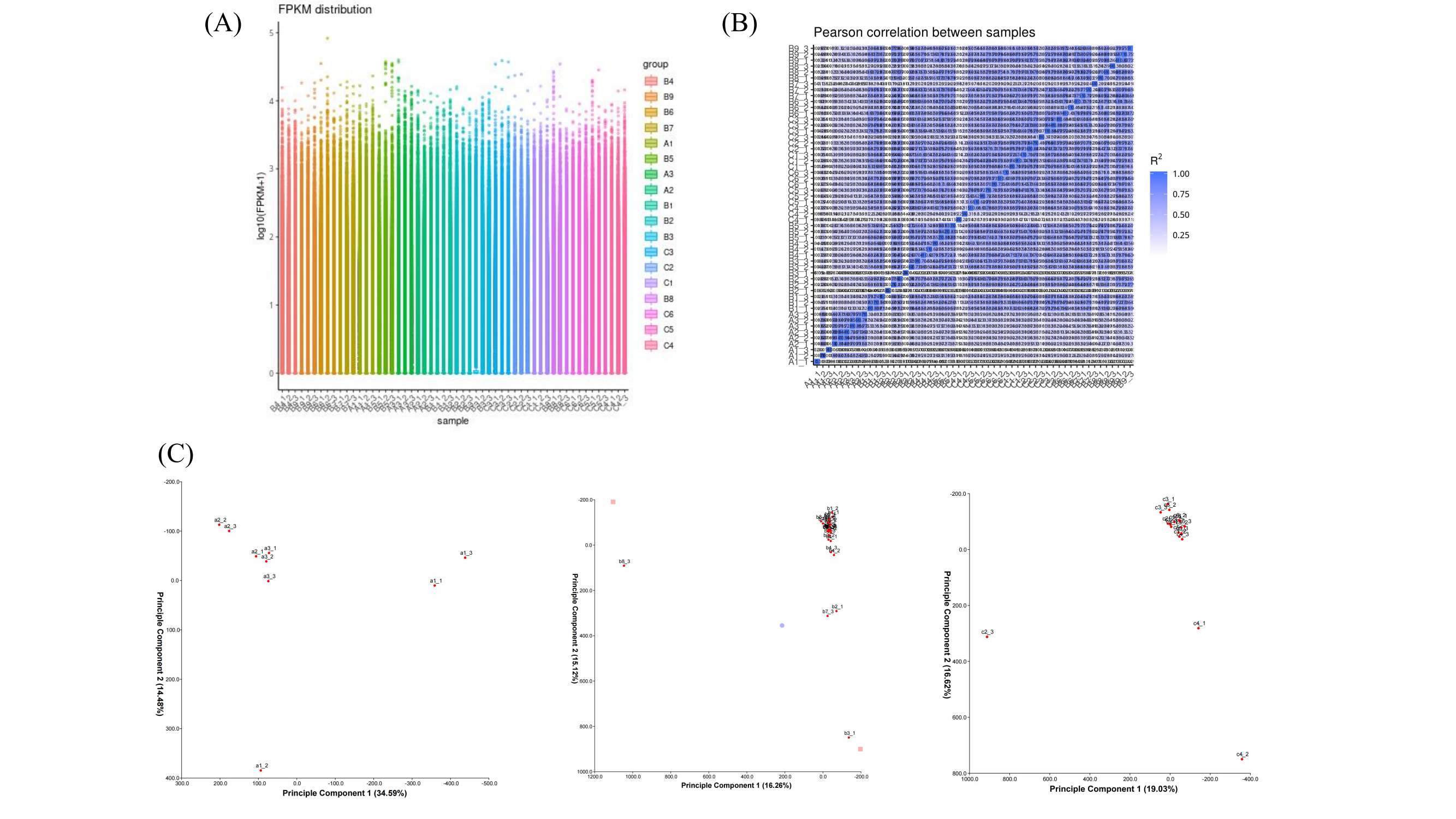

Supplement: Supplementary Figure 2 — Distribution of genes in different samples (A). Correlation between samples (B). PCA of samples from different provinces [file Image_2.tif]

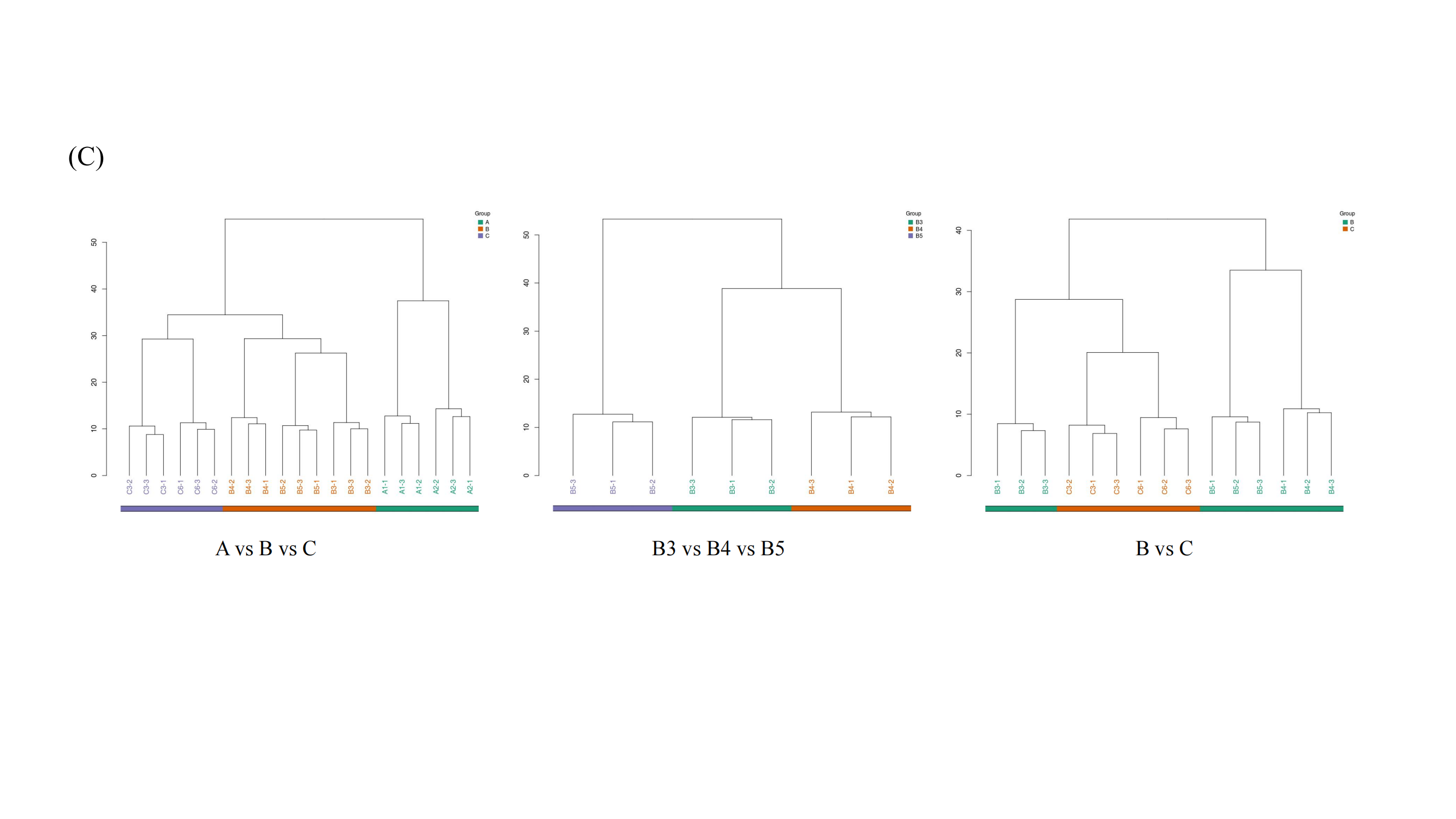

Supplement: Supplementary Figure 3 — Sample hierarchical clustering tree. [file Image_3.tif]

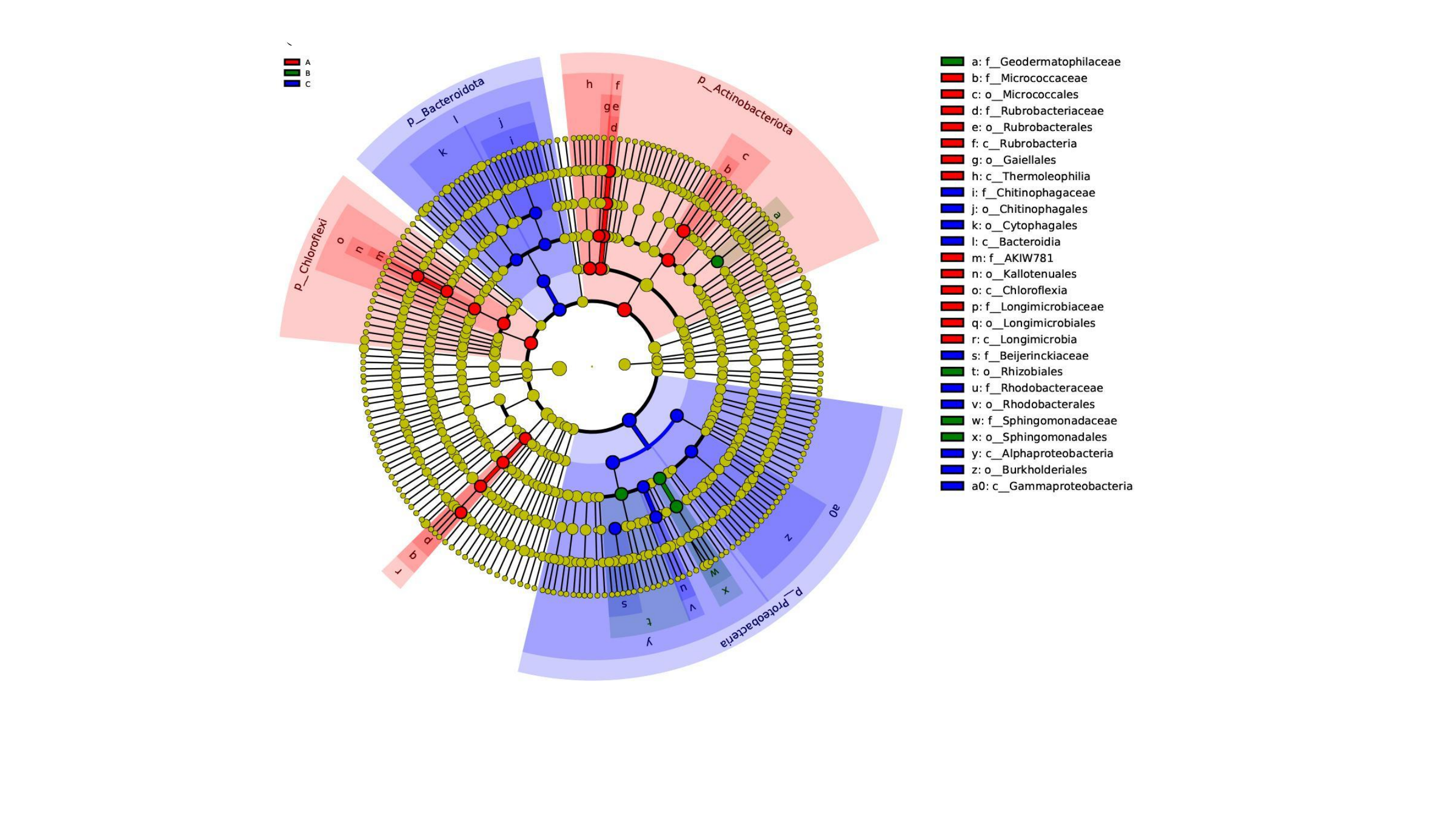

Supplement: Supplementary Figure 4 — LDA distribution histogram of rhizosphere bacterial communities from different producing areas. [file Image_4.tif]

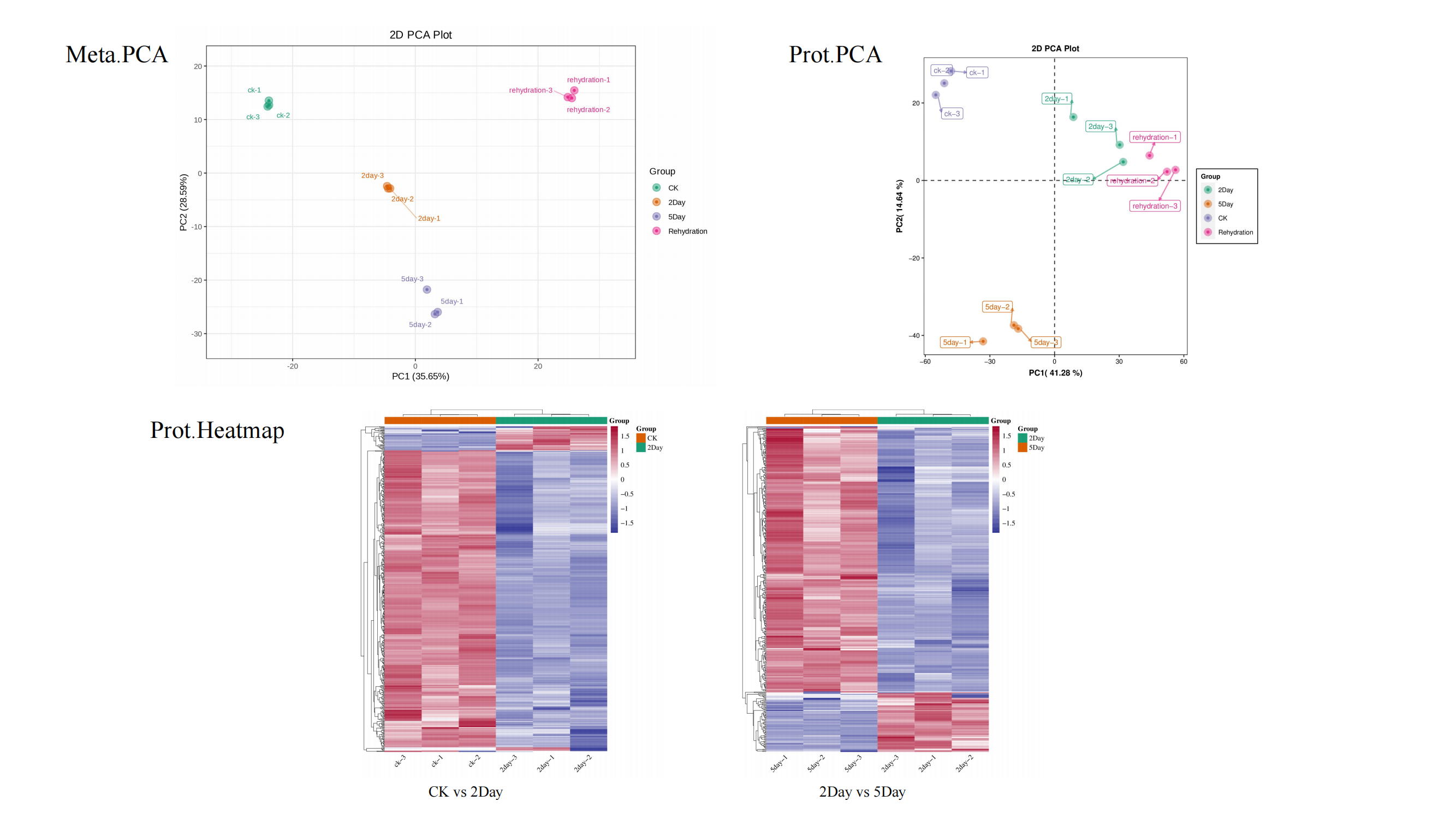

Supplement: Supplementary Figure 5 — PCA analysis (A), and DEPs cluster heat map (D). [file Image_5.tif]

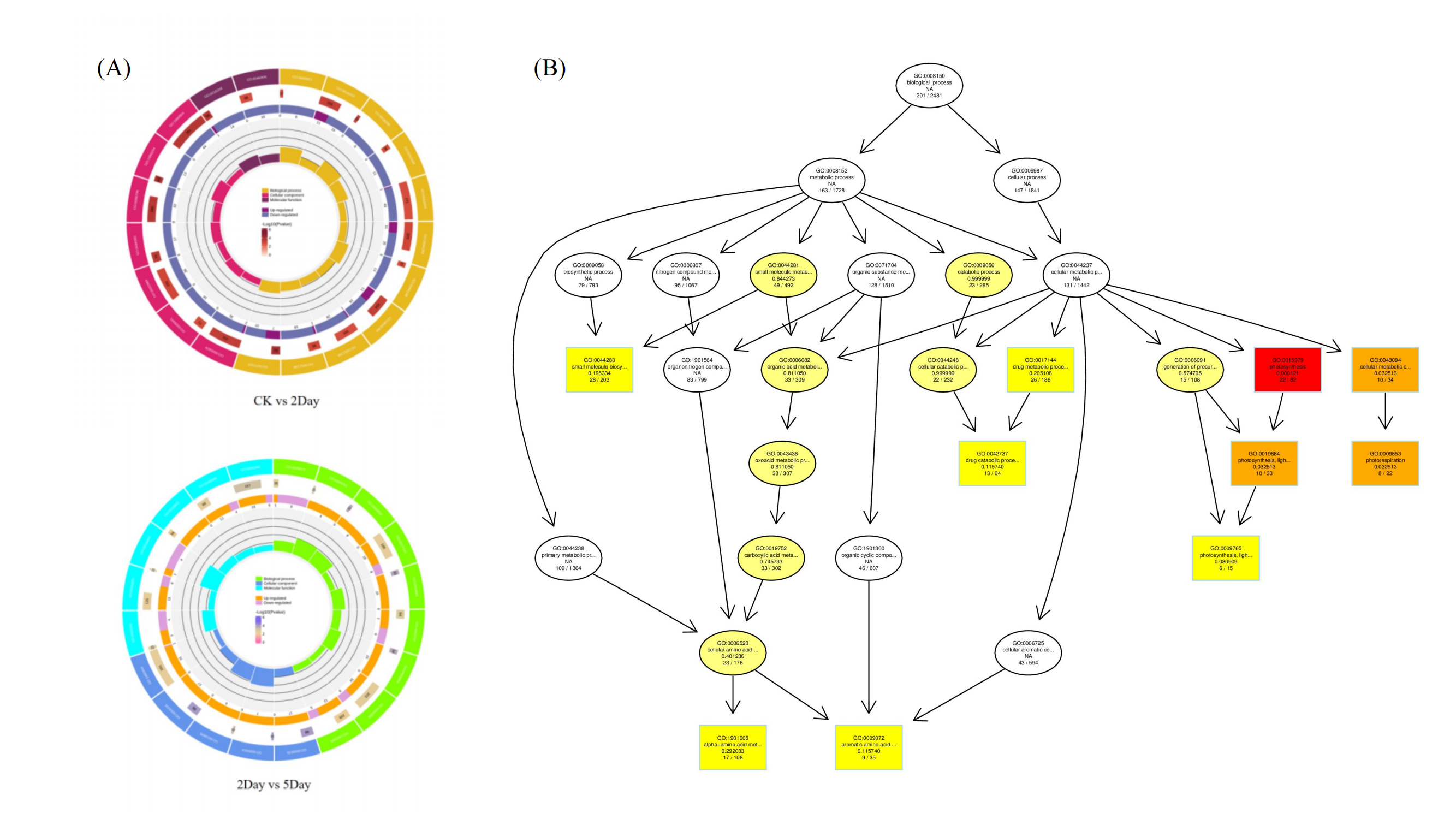

Supplement: Supplementary Figure 6 — GO enrichment circle diagram (A), and directed acyclic graph of enriched GO terms (B). [file Image_6.tif]

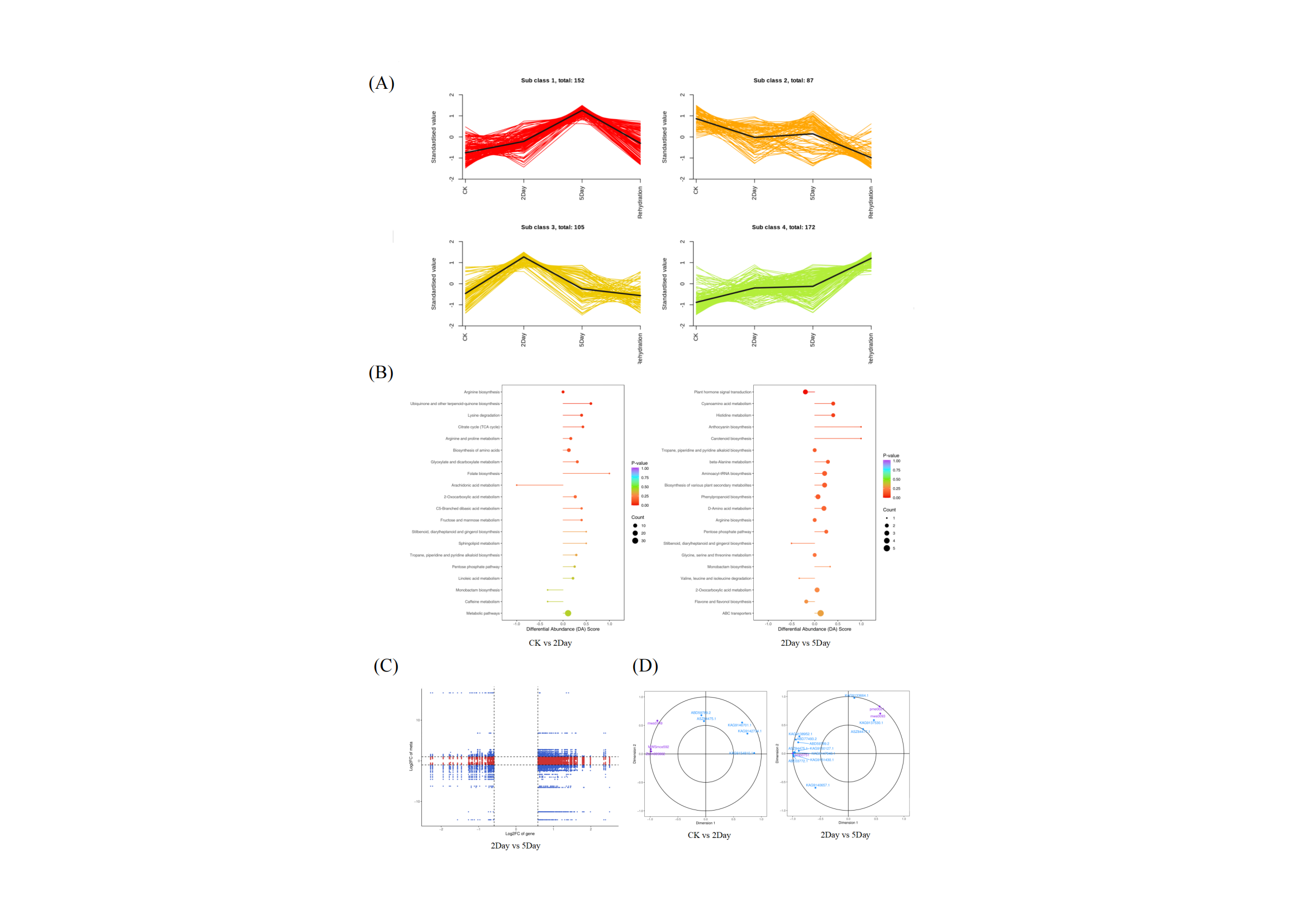

Supplement: Supplementary Figure 7 — K-Means map of differential metabolites (A), metabolite difference abundance score map (B), Correlation analysis of nine quadrants (C), and CCA (D). [file Image_7.tif]

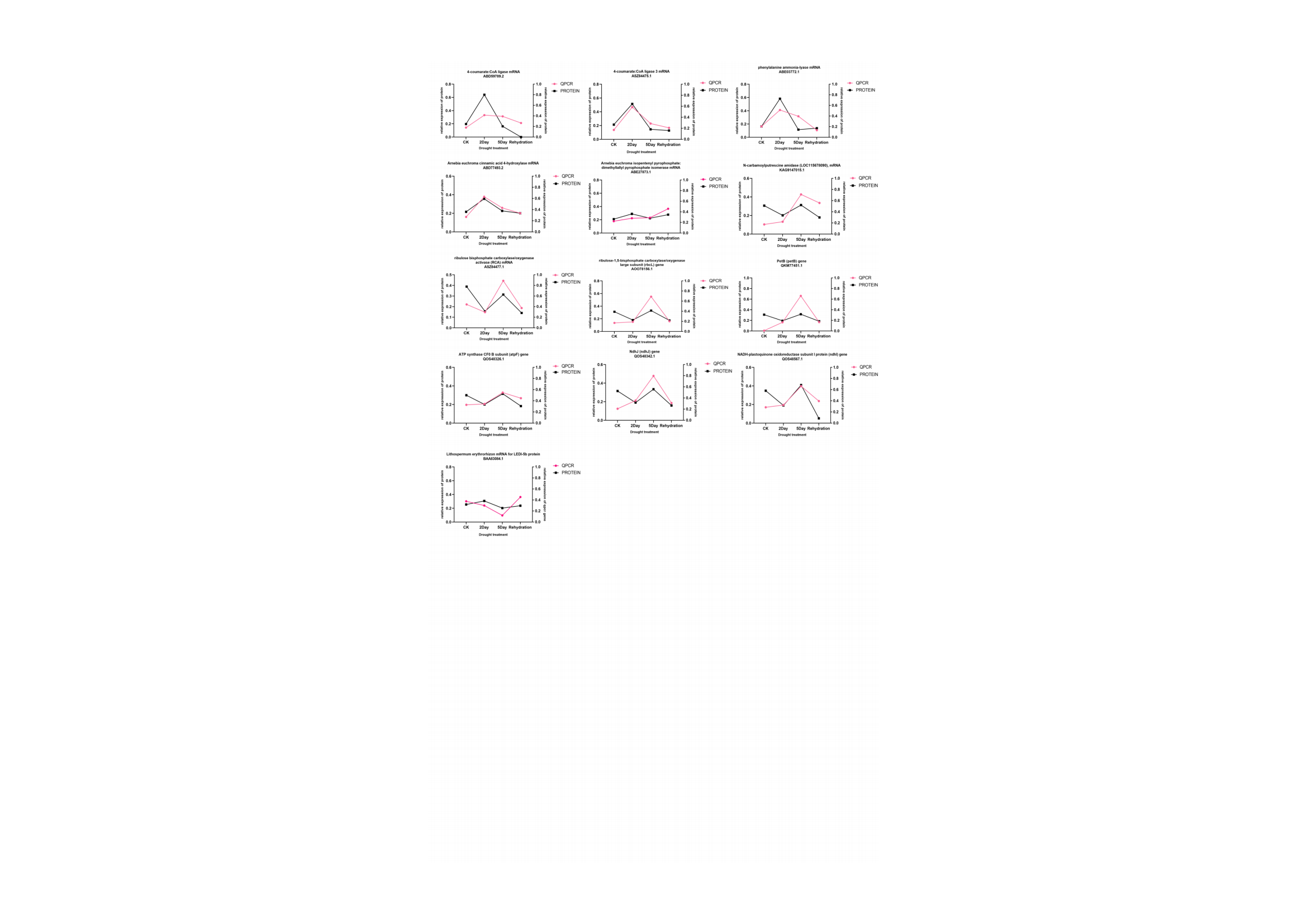

Supplement: Supplementary Figure 8 — Validation of qRT-PCR. The left Y axis is the QPCR coordinate, and the right Y axis is the proteomic coordinate. [file Image_8.tif]

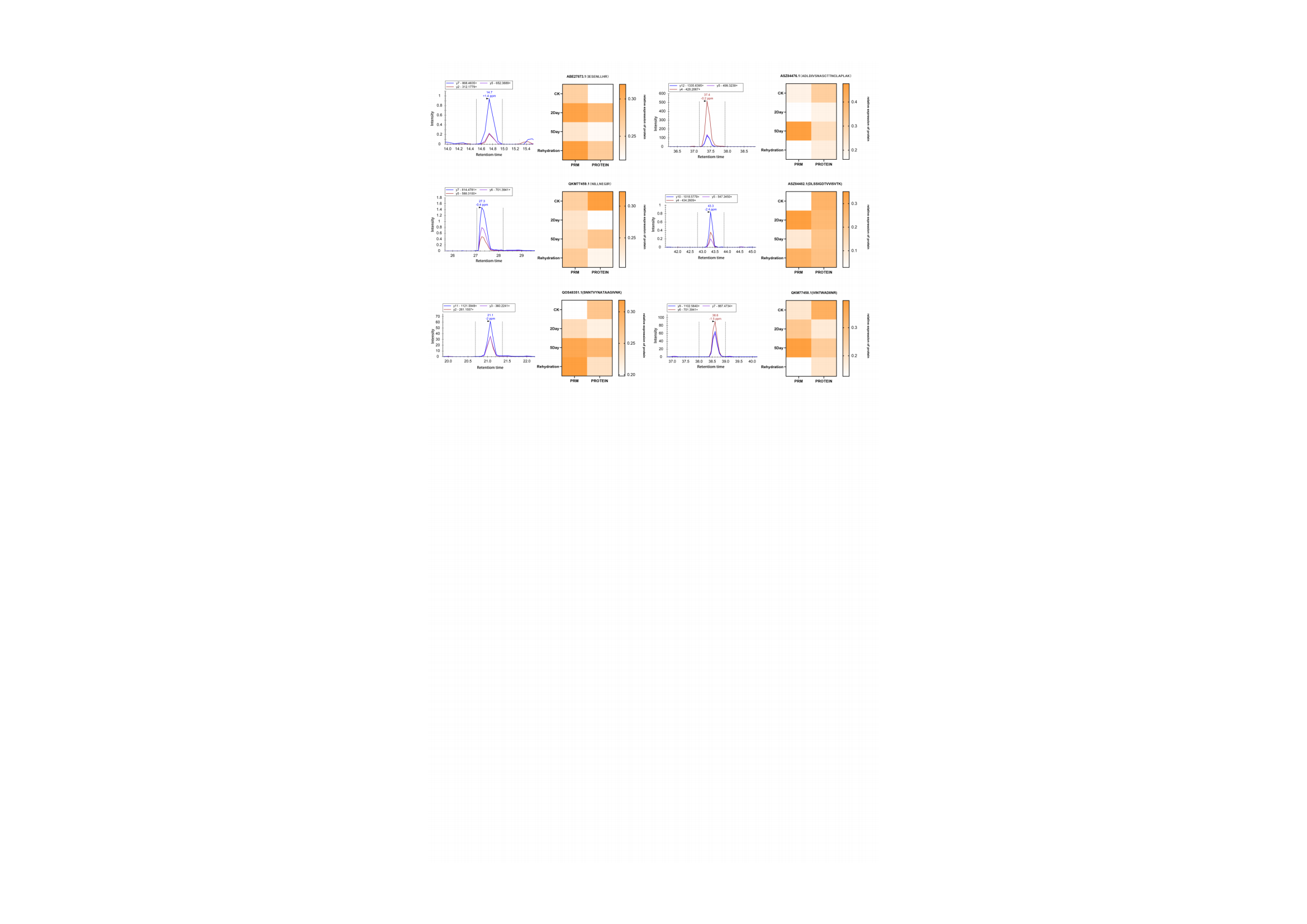

Supplement: Supplementary Figure 9 — Validation of PRM. [file Image_9.tif]
